# RNAi Transfection Results in Lipidome Changes

**DOI:** 10.1002/pmic.201800298

**Published:** 2019-06-13

**Authors:** Cagakan Özbalci, Elisabeth M. Storck, Ulrike S. Eggert

**Affiliations:** ^1^ Randall Centre for Cell and Molecular Biophysics School of Basic and Medical Biosciences King's College London London SE1 1UL UK; ^2^ Department of Chemistry King's College London London SE1 1UL UK

**Keywords:** lipidomics, mass spectrometry, RNA interference (RNAi), transfection reagents

## Abstract

RNAi experiments are ubiquitously used in cell biology and are achieved by transfection of small interfering RNAs (siRNAs) into cells using a transfection reagent. These results in knock‐down of proteins of interest, and the phenotypic consequences are then analyzed. It is reported here that two common RNA interference (RNAi) transfection reagents, DharmaFECT 1 and INTERFERin, in mock transfections using non‐targeting siRNAs, cause alterations in the lipidome of HeLa cells. Some lipids change in response to both, presumably chemically different, transfection reagents, while other lipid species change only in response to one of the reagents. While the functional implications of these lipidomic alterations remain to be investigated, the authors' experiments suggest that it is important to use appropriate mock transfection controls during RNAi experiments, ideally complemented by an orthogonal perturbation, especially when investigating membrane‐associated phenomena.

RNA interference (RNAi) is very commonly used in cell biology to deplete cellular proteins by destroying mRNA encoding a protein of interest. Tens of thousands of articles have been published using RNAi, which complements other methods to perturb protein function such as small molecule treatments and genetic manipulations like CRISPR/Cas9 knockouts.^1^ In an RNAi experiment, small interfering RNAs (siRNAs) are introduced into cells using commercially available transfection reagents (TRs). The composition of these TRs is proprietary; they often include cationic lipids or polymers to facilitate transfer of negatively charged siRNAs across the plasma membrane. Researchers typically empirically determine the best transfection conditions for their cell type and target protein by varying the TR, concentration, and treatment time.

Membrane‐associated proteins play many important roles in cells, and it is becoming increasingly clear that the lipid components of membranes are also important.^2^ Changes in membrane proteins or lipids can have substantial effects on cellular physiology. While lipid biosynthesis is complex and poorly understood, it is clear that cells can respond to and adapt their lipidomes when exposed to different stimuli and many studies have explored lipidome changes in response to protein perturbation by RNAi (e.g., ref. [3]). With so much new cell biology being discovered through RNAi experiments, we wondered if the process of siRNA transfection, in the absence of any protein depletion, affected the lipid composition of cells. This would have profound implications on the experimental design of studies involving membrane proteins and lipids.

We test here the hypothesis that TRs used in siRNA knock down experiments may affect the cellular lipidome. We performed mock siRNA transfections and then analyzed lipidome changes. We chose a very commonly used cultured cell line, HeLa, and two different commonly used TRs (DharmaFECT 1 and INTERFERin) for our analysis. Usually the TR is complexed with siRNA before addition to cells and this complex may have different properties, such as the ability to enter cells, than the TR alone. Therefore, HeLa cells were treated with the TRs alone or complexed with non‐targeting (NT) siRNA for 72 h (Figure 1a,b). To confirm that the experimental conditions result in successful RNAi, we optimized our transfection protocols using target‐specific siRNAs for three cell division‐associated proteins we routinely study in our lab (RACGAP1, Anillin, and CAPZB, Figure S1, Supporting Information).^4^ Immunoblot analyses of total cell lysates illustrate that our protocol achieves penetrant protein knock down and that the knock down efficiency is comparable for both TRs (Figure S1, Supporting Information). Importantly, no cell toxicity was evident at the concentrations of TR used.

**Figure 1:**
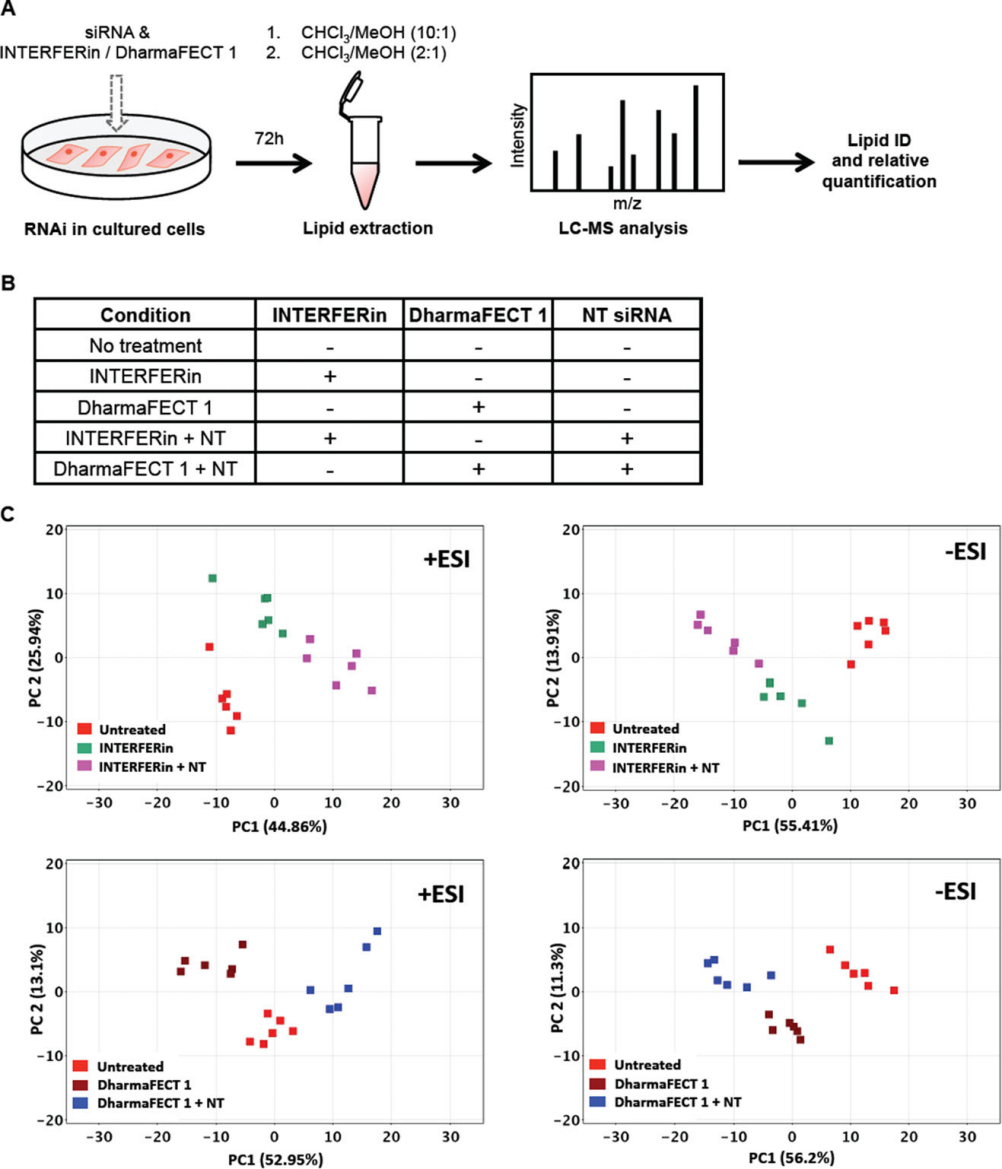
Experimental workflow and conditions used in this study and initial data analysis. A) Cartoon of the workflow used in this study. Cultured HeLa cells were subjected to RNAi for 72 h. Cells were scraped into a micro‐centrifuge tube and subjected to a two‐step chloroform/methanol lipid extraction protocol. Extracts were analyzed by reversed phase liquid chromatography‐mass spectrometry (LC‐MS). MS/MS confirmation or relative quantification was performed to identify lipid species altered in experimental conditions versus untreated control cells. B) The transfection conditions tested in this study. Two different transfection reagents (INTERFERin and DharmaFECT 1) were tested on their own or in combination with non‐targeting siRNA (NT). Untreated cells served as a control. C) Principal component analysis representing the separation of untreated HeLa cells from HeLa cells treated with INTERFERin (top panels) or DharmaFECT 1 (bottom panels) and TRs plus NT siRNAs in positive (left panels) and negative (right panels) modes. PCA plots were generated by nontargeted analysis with one‐way ANOVA *p* (Corr) (cut‐off = 0.05) test.

With our optimized transfection protocol in hand, we performed siRNA transfections and subsequently extracted lipids for lipidomic analysis. Samples were prepared in two independent experiments with three replicates of each condition. First, cells grown in 6‐well dishes were washed with cold PBS, scraped, and transferred to a microfuge tube. An aliquot was taken for protein concentration determination. Because it is difficult to accurately determine overall lipid concentrations, samples for lipidomic analysis were normalized based on protein concentration. The cell samples were pelleted, re‐suspended in PBS, and spiked with an internal lipid standard (d5‐TG ISTD Mix I) for quality control of the extraction process and as a reference for lipidomics. To obtain both hydrophobic and amphiphilic lipids, we used a 2‐step extraction method.^5^ In the first step, we added chloroform:methanol (10:1, v/v) to the cell suspensions and recovered the lower organic phase. The upper aqueous phase was re‐extracted with chloroform:methanol (2:1, v/v) (Figure 1A). The lipid extracts were evaporated under a stream of nitrogen, reconstituted in loading buffer (isopropanol:water:acetonitrile, 2:1:1) and analyzed by reversed phase liquid chromatography‐mass spectrometry (RP LC‐MS).

We analyzed samples by RP LC‐MS analysis using a 1290 Infinity UHPLC system coupled to a 6550 iFunnel quadrupole time‐of‐flight mass spectrometer (LC QTOF MS) from Agilent Technologies. Extracted lipids were separated on an Acquity UPLC CSH C18 column (100 × 2.1 mm, 1.7 μm) (Waters) and features were detected in negative and positive modes. Analytical conditions and mass spectrometric parameters were adapted from Cajka and Fiehn^6^ with minor modifications (see Experimental Methods in SI). We injected samples in random order to avoid systematic contamination and included controls of TR only. The features detected in these samples (due to TRs and MS solvents) (Figure S2 and Table S1, Supporting Information) were removed from subsequent analyses.

We first performed a principal component analysis (PCA) on our data sets to determine overall differences. Interestingly, each set of conditions with both TRs (untreated HeLa, HeLa treated with TR, and HeLa treated with TR complexed to NT siRNA) clustered separately, both in positive and negative modes, supporting our hypothesis that TRs may impact cellular lipidomes (Figure 1C). Unexpectedly, the presence of NT siRNAs also affected lipidomes, with clear separate clusters for these conditions. The only variable in these experiments was the presence or absence of NT siRNA. The function of a TR is to complex siRNA and carries it across the plasma membrane, and this is what it has been optimized for. Therefore, it is possible that the cellular uptake of the complex is more efficient than that of the TRs alone and they may access cellular membrane structures differently. Our analysis cannot differentiate between these possibilities as it captures the total cellular lipidome and does not resolve subcellular compartments. It is also possible that the NT siRNAs target unknown miRNAs or have off‐target effects even though they have been designed to not target human mRNAs, which could be addressed by analyzing NT siRNAs with different sequences. More broadly, there remains much to be learned about the molecular mechanisms of siRNA transfection and subsequent dissociation of the siRNA from the TR, which may be needed for the silencing reaction to occur. It has been challenging to address these questions in part due to the proprietary composition of the TRs and in part due to a lack of experimental techniques that would allow a step by step analysis.

Having shown that both TR and TR plus NT siRNA resulted in overall changes to the lipidome, we next determined if specific lipid families or species were changed. We applied untargeted analysis followed by a complementary targeted data analysis for common lipid classes and species. We used an in‐house database that includes MS/MS data correlated with retention times to identify lipid species (e.g., Figure S3, Supporting Information) and analyzed the data as reported in the Experimental Methods in the Supplementary Materials. Most lipid classes were broadly unaffected. There were no significant changes to lipid families after INTERFERin treatment, while TGs, DGs, and FAs changed slightly but significantly after DharmaFECT 1 plus NT siRNA treatment (Figure 2). This is consistent with a large body of literature based on the assumption that control siRNA transfections do not generally grossly perturb cellular phenotypes.

**Figure 2:**
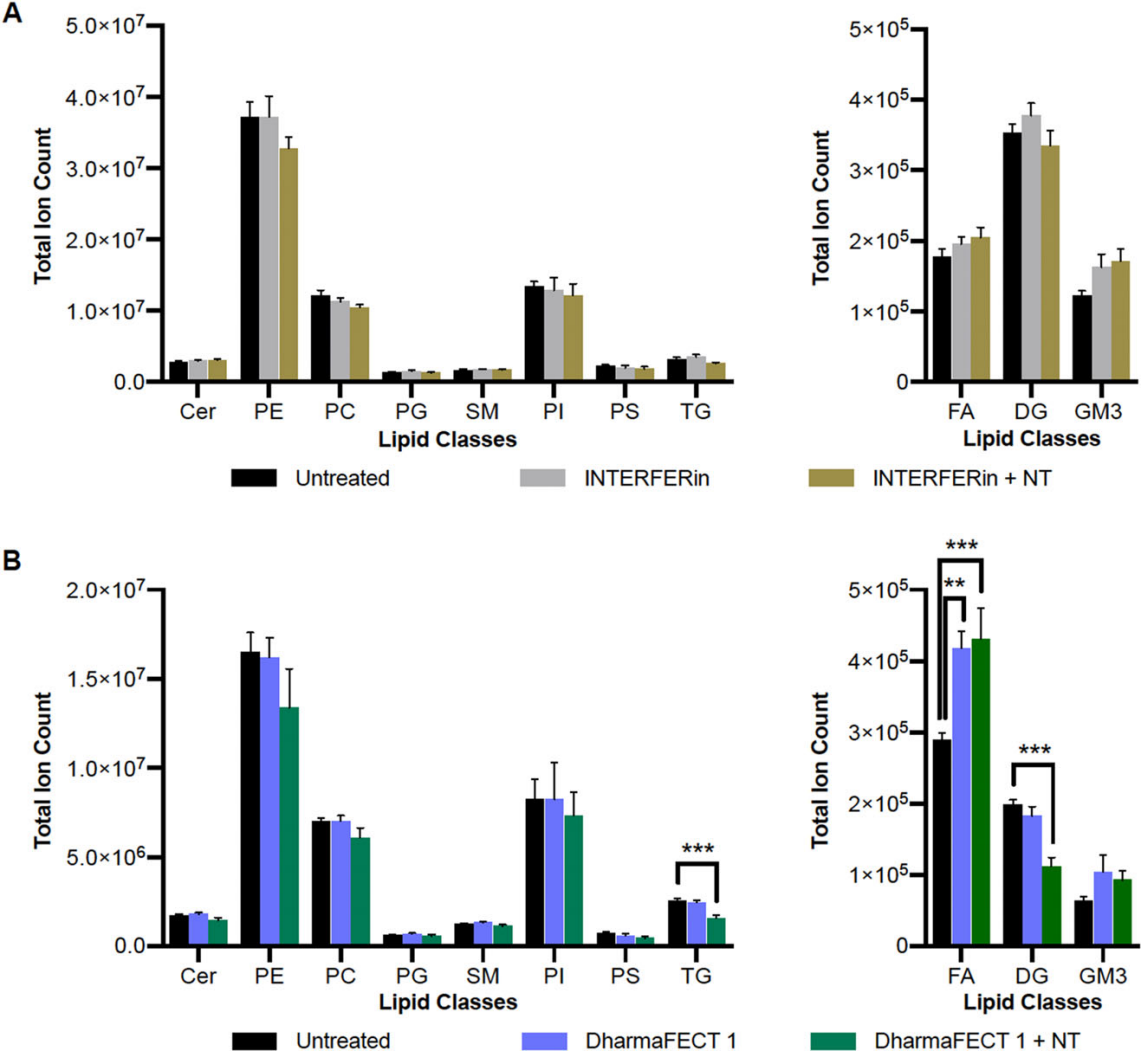
Targeted lipid class analysis of untreated HeLa cells and HeLa cells treated with transfection reagents. Comparison of major lipid class raw total intensities in A) untreated, INTERFERin and INTERFERin plus NT siRNA‐treated HeLa and B) untreated DharmaFECT 1 and DharmaFECT 1 plus NT siRNA‐treated HeLa. Less abundant species are shown in right panels. Error bars indicate +/‐ SEM (*n* = 6). Significance analysis and multiple comparisons were performed by ANOVA and Dunnet tests with *p*‐value style < 0.0021(**), < 0.0002(***). Cer, ceramide; DG, diacylglycerol; FA, fatty acid; GM3, Ganglioside GM3; PC, phosphatidylcholine; PE, phosphatidylethanolamine; PG, phosphatidylglycerol; PI, phosphatidylinositol; PS, phosphatidylserine; SM, sphingomyelin; TG, triacylglycerol.

Interestingly, we found numerous individual lipid species changed in response to TR treatments (Figure 3; see Figures S4 and S5, Supporting Information, for graphs showing fold changes for all species analyzed in the targeted analysis and Tables S2 and S3, Supporting Information, for details on the changed species). As noted during PCA analysis TR plus NT siRNA treatment differed from TR alone and the effects on specific species we observed were generally stronger in the TR plus siRNA treatments (Figure 3). Remarkably, three lipid species changed in response to treatment with both TRs (Figure 3 and Table S1, Supporting Information): FA 24:5 and 2 PG species (out of many PG species detected): PG 22:5 22:6 and PG 16:1 22:6.

**Figure 3:**
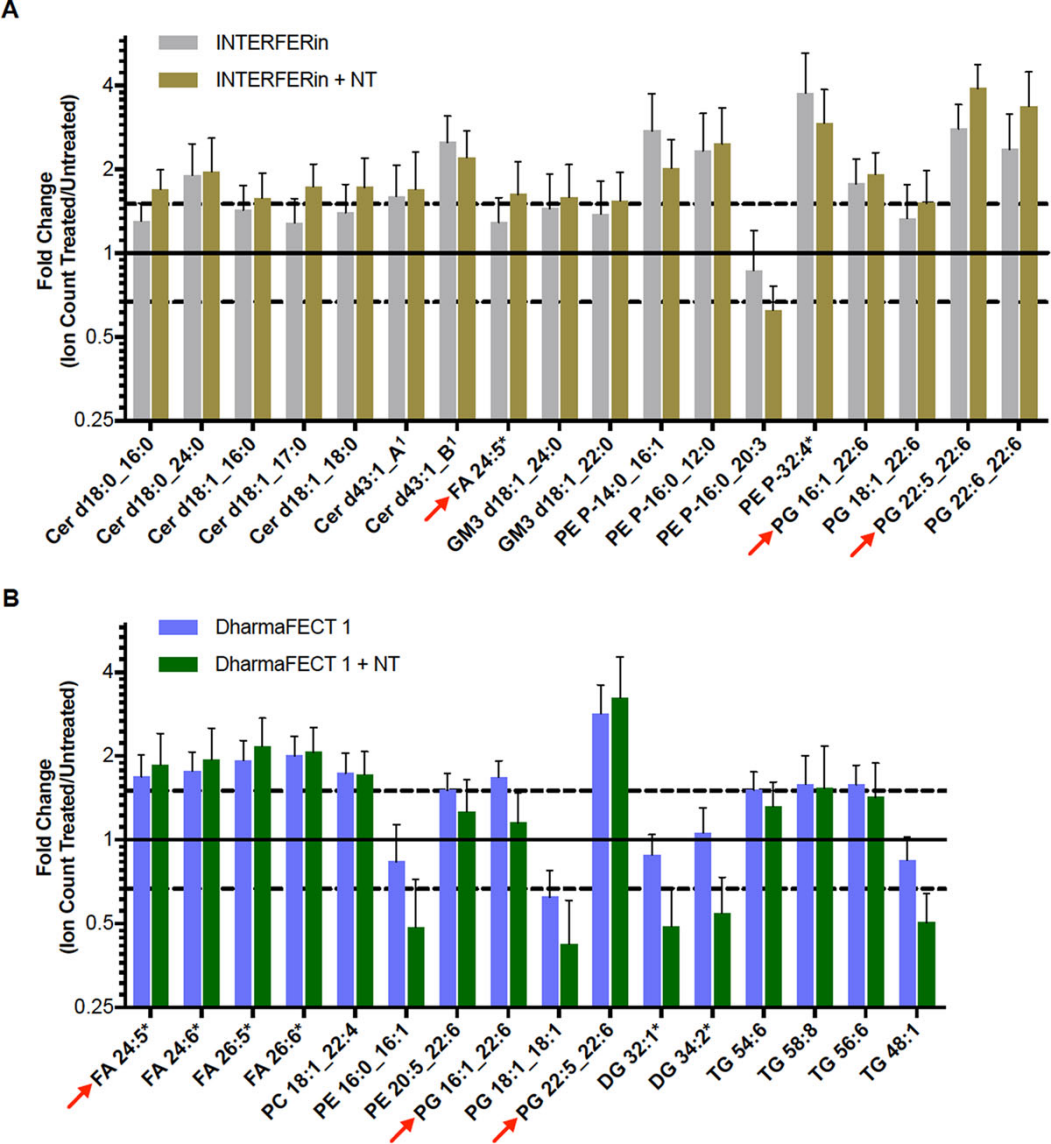
Lipid species altered in TR‐treated compared to untreated HeLa cells. A) Significantly altered lipid species in INTERFERin and INTERFERin plus NT siRNA‐treated HeLa cells. B) Significantly altered lipid species in DharmaFECT 1 and DharmaFECT 1 plus NT siRNA‐treated cells. Six independent samples from two separate experiments were run for each condition. Statistical analysis was performed in Mass Profiler Professional as detailed in the Experimental Methods and ion counts of altered species were re‐extracted in Profinder. Fold change was calculated by normalizing the mean ion count of treated samples (*n* = 6) to the mean ion count of untreated samples (*n* = 6). Error bars represent SD. Lipid species indicated with red arrows are increased in both INTERFERin and DharmaFECT 1 treated cells. Lipid species indicated by black asterisks (*) were predicted by METLIN metabolomics, LipidBlast or LIPID MAPS databases using accurate mass and were not confirmed by MS/MS. 1) Two species of Cer d43:1 were detected but side‐chain composition could not be resolved by MS/MS. See Figures S4 (DharmaFECT 1) and S5 (INTERFERin), Supporting Information for graphs showing all analyzed lipid species. Cer, ceramide; DG, diacylglycerol; FA, fatty acid; GM3, Ganglioside GM3; PC, phosphatidylcholine; PE, phosphatidylethanolamine; PG, phosphatidylglycerol; TG, triacylglycerol.

Taken together, our data show that cells can adapt their lipidomes in response to treatment with TRs. Several different responses are possible and may occur in parallel. First, cells could alter their lipid metabolism to adapt to changes in cellular membrane properties, such as membrane fluidity or permeability changes, caused by TR molecules inserting into or interacting with membrane components. It is possible that the three lipids that change in all TR samples are a consequence of such an adaptation since these lipids change independently of the chemical identity of the transfection reagent. Second, it is conceivable that TR components could be metabolized and/or be incorporated into lipid structures. It is difficult to evaluate this possibility without knowing the proprietary composition of the TRs, but we did not see any large peaks corresponding to unnatural lipids, suggesting this effect is limited. Third, the overall health of the cell may be affected after transfection and responses such as apoptosis could be triggered. Our cells looked healthy and grew well and we saw no obvious signs of TR toxicity. However, we did observe an increase in TGs and DGs after DharmaFECT 1 plus NT siRNA treatment, a lipid change also observed during cell death.^7^ It is possible that subtle changes towards apoptotic initiation occurred in this treatment.

The functional implications of the lipid changes we report here require further examination, and there may be many cases where there is no effect on the biology under investigation. However, common sense would suggest that RNAi experiments are always accompanied by controls where a mock transfection is performed in parallel, as is already being done by many careful investigators. In cases where a phenotypic consequence of lipid alterations is suspected, we would suggest using multiple TRs and ideally confirming results by an orthogonal method such as shRNA (which can be used to create stable cell lines) or CRISPR/Cas9.

## Conflict of Interest

The authors declare no conflict of interest.

## Supporting information

Supporting Information

Supporting Information

Supporting Information

Supporting Information

Supporting Information

Supporting Information

Supporting Information

Supporting Information

Supporting Information
