# Supplementary material for: RNAi Transfection Results in Lipidome Changes
Source: Proteomics. 2019 Jun 13;19(13):1800298. doi: 10.1002/pmic.201800298 (PMC6617754; doi:10.1002/pmic.201800298)
Supplement: Supplementary file 1 — Supporting Information [file PMIC-19-na-s001.docx]

**Supplementary Information & Experimental Methods**

**RNAi transfection results in lipidome changes**

Cagakan Özbalci^1, †^, Elisabeth M. Storck^1, †^ and Ulrike S. Eggert^1, 2,^ *

^1^ Randall Centre for Cell and Molecular Biophysics, School of Basic and Medical Biosciences, King’s College London, London, SE1 1UL, UK

^2^ Department of Chemistry, King’s College London, London, SE1 1UL, UK

**Supplementary Figure and Table Legends**

**Supplementary Figure 1. RNAi using INTERFERin and DharmaFECT 1 transfection reagents result in comparable and penetrant protein knockdown.** HeLa cells were subjected to RNAi using siRNA targeting RACGAP1, Anillin or CAPZB for 72h (‘+’ lanes). Non-targeting siRNA was used as a control (‘-‘ lanes). Transfections were performed using INTERFERin or DharmaFECT 1 transfection reagents (see experimental methods for detailed protocol). Immunoblot analyses of whole cell lysates using the indicated antibodies was performed to assess protein knock-down.

**Supplementary Figure 2.** Base peak (500-1400) chromatograms (BPC) of loading buffer (LB), LB + DharmaFECT 1 and LB + INTERFERin in A) positive and B) negative modes. The injection volume for positive mode was 1 µL and 3 µL for negative mode.

**Supplementary Figure 3.** MS/MS fragmentation matching of lipid species with fold change (FC)>1.5 compared to untreated HeLa cells. LipidBlast in silico tandem mass spectrometry database was employed for the annotation.

**Supplementary Figure 4**: Quantitative comparison of detected lipid species between untreated HeLa, HeLa treated with DharmaFECT 1 and HeLa treated with DharmaFECT 1 and NT siRNA. Averages of extracted intensities of individual lipid species from biological replicates were used to generate the bar plots. A and B indicate isomers with different retention times. Error bars are +/- standard deviation (n = 6).

**Supplementary Figure 5**: Quantitative comparison of detected lipid species between non-treated HeLa, HeLa treated with INTERFERin and HeLa treated with INTERFERin and NT siRNA. Averages of extracted intensities of individual lipid species from biological replicates were used to generate the bar plots. A and B indicate isomers with different retention times. Error bars are +/- standard deviation (n = 6).

**Supplementary Table 1** List of the detected features originating from the transfection reagents. Features detected in negative and positive modes are listed on the left and right tables, respectively.

**Supplementary Table** **2** List of the altered lipid species in INTERFERin/ INTERFERin plus NT siRNA HeLa cells relative to untreated HeLa cells. Species in **bold letters** are increased in both TR treatments.

**Supplementary Table 3** List of the altered lipid species in DharmaFECT 1/DharmaFECT 1 plus NT siRNA treated HeLa cells relative to untreated HeLa cells. Species in **bold letters** are increased in both TR treatments.

**Experimental Methods**

**Reagents**

Dulbecco’s modified Eagle’s medium (DMEM, high glucose, GlutaMAX™, pyruvate), fetal bovine serum (FBS) and Opti-MEM™ low serum media were purchased from Gibco®. Penicillin-streptomycin (P/S) was purchased from Merck.

LC-MS grade water, organic solvents and additives were purchased from Honeywell, Fisher Scientific or Merck. Lipid standards were purchased from Avanti Polar Lipids.

INTERFERin® (cat. no. 409-10, lot 08INF0311L1) was purchased from Polyplus-transfection. DharmaFECT 1 (cat. no. T-2001-02, lot 00699995) and siRNA were purchased from Dharmacon (Horizon Discovery). Non-targeting (NT) control siRNA and siRNA targeting RacGAP1 and Anillin were used as a pool of 4 oligos and siRNA targeting CAPZB was an individual oligo (see table below for sequences). siRNAs were reconstituted at a concentration of 20 µM in 1x siRNA Buffer (Dharmacon).

| **Gene** | **Cat. No.** | **siRNA Sequences** |
| --- | --- | --- |
| NT control | D-001810-10  (ON-TARGETplus SMARTpool) | UGGUUUACAUGUCGACUAA, UGGUUUACAUGUUGUGUGA, UGGUUUACAUGUUUUCUGA, UGGUUUACAUGUUUUCCUA |
| RacGAP1 | M-008650-00 (siGENOME SMARTpool) | CAAAUUAUCUCUGAAGUGU, CCACAGACACCAGAUAUUA, GAACAUCAGCUUCUCAAGA, GUAAUCAGGUGGAUGUAGA |
| Anillin | M-006838-03 (siGENOME SMARTpool) | GGAGAUGGAUCAAGCAUUA, GGAUAAAUCUGGCUAAUUG,  ACGCAACACUUUUGAAUUA, ACAGAAAAGGUGACCGAAA |
| CapZB | D-011990-01  (siGENOME individual) | GAAGUACGCUGAACGAGAU |

**Cell Culture and RNAi**

HeLa cells were cultured in DMEM supplemented with 10% FBS and P/S (100 U/L, 100 µg/L) and maintained in a humidified incubator (37°C, 5%CO_2_).

Cells were plated at a density of 100,000 cells/well in a 6-well dish the day prior to transfection. Prior to siRNA transfection, growth medium on cells was replaced with P/S-free medium (2 mL/well).

Transfection with INTERFERin:

Quantities given are for transfection of one well of a 6-well plate. Opti-MEM™ (500 µL), siRNA (5 µL, 20 µM, 40 nM final) and INTERFERin (7.5 µL) were mixed and incubated at room temperature for 10 min. The solution was added to cells. Growth medium was replaced after 6h with medium containing antibiotics.

Transfection with DharmaFECT 1:

Quantities given are for transfection of one well of a 6-well plate. Solution A was prepared by adding siRNA (5 µL, 20 µM, 40 nM final) into 250 µL Opti-MEM™. Solution B was prepared by adding DharmaFECT 1 (2 µL) into 250 µL Opti-MEM™. After incubation at room temperature for 5 min solution A and B were combined and incubated a further 20 min before addition to cells. Growth medium was replaced after 6h with medium containing antibiotics.

For transfection-reagent only samples siRNA reagent was omitted, and for untreated samples Opti-MEM™ (500 µL) only was added to cells.

**Immunoblot analysis**

The following antibodies and dilutions were used: goat anti-RACGAP1 (1:1000, EB05315, Everest Biotech), mouse anti-Anillin (1:1000, Abcam, ab211872), rabbit anti-CAPZB (1:500, ab175212, Abcam), mouse anti-GAPDH (1:10000, 60004-1-Ig, Proteintech®), sheep anti-tubulin (1:1000, ATN02, Cytoskeleton Inc). AffiniPure donkey HRP-conjugated secondary antibodies were purchased from Jackson ImmunoResearch and used at 1:5000 dilution.

Samples for immunoblot blot analysis were prepared in 6-well dishes and processed 72h post-siRNA treatment. Cells were washed with PBS (2x) and denatured in 500 μL lysis buffer (3% SDS, 15% glycerol, 94 mM Tris pH 6.8) by passing several times through a 25G needle and boiling (100°C, 10 min). Protein concentrations were determined using the Pierce™ BCA Protein Assay Kit (Thermo Scientific) according to the manufacturer’s instructions. Lysates were supplemented with DL-Dithiothreitol (100 mM final) and bromophenol blue (0.01% final). 15 µg of protein lysate was resolved by SDS-PAGE on NuPAGE™ 4-12% Bis-Tris gels in NuPAGE™ MOPS SDS running buffer and subsequently transferred to a nitrocellulose membrane (0.2 µm, Bio-Rad) via wet-tank transfer. Membranes were incubated in blocking buffer (5% non-fat milk in PBS with 0.01% Tween-20 (PBS-T)) for 1 hour at room temperature, followed by incubation with primary antibody in blocking buffer at 4°C overnight. Membranes were washed with PBS-T (30 min) and incubated with HRP-conjugated secondary antibody in blocking buffer for 2h at room temperature. After washing as before, membranes were developed with Amersham™ ECL™ Prime Western Blotting Detection Reagent (GE Healthcare) and imaged on a G:BOX Chemi XR 5 (SynGene) chemiluminescent detection system. Signal was quenched by incubating the membranes in blocking buffer with 1% sodium azide for 1h at room temperature and the membrane re-probed as above for GAPDH or tubulin as a loading control.

**Lipid extraction**

Samples were processed 72h post-RNAi treatment. Samples were prepared in two independent experiments with three replicates of each condition (totaling 6 samples per condition). Cells were washed with cold PBS (2x), scraped into 500 µL cold PBS on ice and transferred to a micro-centrifuge tube. An aliquot (10%) was taken for protein concentration determination. The remaining sample was snap-frozen in liquid nitrogen and stored at -80°C until further processing. For protein concentration determination cells were lysed in M-PER™ Mammalian Protein Extraction Reagent (Thermo Scientific) and protein content determined using the Pierce™ BCA Protein Assay Kit (Thermo Scientific) as per the manufacturer’s instructions. 60 µg or 100 µg of protein per sample were used for lipid extraction for DharmaFECT 1 and INTERFERin sample sets, respectively. Cells were pelleted (500xg, 5 min, 4°C), and subsequently re-suspended in 200 µL of ice-cold PBS and spiked with internal standard d5-TG ISTD Mix I. Lipid were extracted by a 2-step extraction method [1]. 990 µL of ice-cold extraction solvent A (chloroform-methanol, 10:1, v/v) was added, the sample vortexed for 30 sec followed by incubation on a rotating wheel for 1h at 4°C. Phases were separated by centrifugation (9000x*g*, 2 min, 4°C) and the lower organic phase recovered. The upper aqueous phase was re-extracted with 1 mL ice-cold extraction solvent B (chloroform-methanol, 2:1, v/v) as above. The lipid extracts were evaporated under a stream of nitrogen and stored at -20°C. Before analysis, lipid extracts were reconstituted in 100 µL loading buffer (isopropanol:water:acetonitrile, 2:1:1). For positive mode, 2.5 µL and negative mode, 12 µL of sample were injected in randomized order. Additionally, transfection reagents were diluted in loading buffer (1:100) and 1 µL and 3 µL were injected for positive and negative mode, respectively.

**Liquid chromatography-mass spectrometry analysis**

Reversed phase liquid chromatography-mass spectrometry (RP-LC-MS) analysis was performed on a 1290 Infinity UHPLC system coupled to a 6550 iFunnel quadrupole time-of-flight mass spectrometer (LC-QTOF-MS) from Agilent Technologies. Extracted lipids were separated on an Acquity UPLC CSH C18 column (100 × 2.1 mm, 1.7 µm) (Waters) and features were detected with negative and positive modes. Analytical conditions and mass spectrometric parameters were adapted from Cajka and Fiehn with minor modifications [2]. Flow rate of the analytical gradient was adjusted to 0.600 mL/min using by mobile phase A (water:acetonitrile, 4:6, v/v) and mobile phase B (isopropanol:acetonitrile, 9:1, v/v). For positive mode 10 mM ammonium formate and 0.1 % formic acid were used as modifiers whereas 10 mM ammonium acetate was added to the mobile phases for negative mode.

Gradient elution was carried out as follows: 0−2 min: 15-30% B; 2-2.5 min: 30-48% B; 2.5−11 min: 48-82% B; 11-11.5 min: 82-99%B; 11.5-14.50: 99% B. The gradient was returned to initial conditions over 0.5 min and the columns equilibrated for 3 minutes before subsequent runs. Between injections a 100% isopropanol needle wash was performed.

Electrospray parameters were set as follows: gas and sheath gas temperature, 200 °C; drying gas and sheath gas flow, 14 L/min-11L/min; nebulizer pressure, 35 psig; capillary and nozzle voltage, 3000 and 1000V. MS-TOF fragmentor and Oct 1 RF Vpp radio voltage were set to 350 and 750 V respectively.

The QTOF was calibrated and operated in the extended dynamic range mode (∼2 GHz) in the mass range 50 to 1700 *m/z*. Spectra were acquired in centroid mode with an acquisition rate of 2 spectra/s from 50 to 1700 *m/z*. LC-MS/MS data was acquired in pooled quality control samples by auto-MS/MS analysis (data-dependent) at a rate of 2 spectra/s and 4 spectra/s for MS1 and MS/MS acquisitions. Fragmentation was triggered if the precursor reached 200 counts and maximum precursors per cycle was set to 3. In order to increase the fragmentation quality, background ions were added to exclusion list. Collision energy was adjusted to 25 eV and -20 eV for positive and negative modes. MS/MS isolation width for precursors was selected as narrow (1.3 *m/z*).

**Data processing and statistics for untargeted analysis**

Data pre-processing was carried out using the Mass Hunter Profinder Software (version B.08.00) from Agilent Technologies. For non-targeted feature extraction, the “Batch Recursive Feature Extraction for Small Molecule” option was selected. H^+^, Na^+^ and NH_4_^+^ adducts were selected for positive mode and H^−^, CH_3_COO^−^ and HCOO^−^ adducts were selected for negative mode. Retention time (RT) span was set to 0.300 min and mass tolerance was set to 20ppm + 2mDA for the compound binning and alignment parameters. Features over 20% of saturation limit were excluded from the dataset. Features originating from transfection reagents and solvents were removed from the dataset in order to perform comparable analyses.

Mass Hunter Profinder exports “compound exchange format” (*.CEF) files for each sample, which were imported to Mass Profiler Professional for further statistical analysis. All replicates (n = 6) of each condition were grouped and normalized by quantile algorithm. In order to improve our reported data, we applied a two-step quality control process before carrying out the statistical analysis. In the first step, features which did not exist in at least 80% of samples in any 1 out of 3 conditions (i.e. untreated, TR and TR plus NT) were excluded. In the next step, we filtered the features according to their coefficient of variation. Features which satisfied CV < 25% within 2 out of 3 conditions qualified for the statistical analysis. Remaining features were subjected to statistical analysis by ANOVA (p<0.05) with post-hoc Tukey HSD test for each group. The FDR (Benjamini Hochberg) correction with asymptotic p-value computation was used for the multiple testing correction with fold change analysis (FC>1.5) and all TR and TR+NT conditions were evaluated against untreated HeLa samples.

The annotation of the majority of the altered features was accomplished by MS/MS fragmentation. LipidBlast [3] and LIPID MAPS and METLIN Metabolomics databases were employed for matching the lipid fragments. Accurate mass (5<ppm), retention time and pattern of the adducts were taken in to account for the annotations where MS/MS analysis was unavailable. Raw intensities of significantly altered features were extracted again and inspected in Profinder. To calculate fold change (FC) values, the mean ion count (n=6) of TR or TR + NT samples were normalized against the mean ion count (n=6) of untreated samples. Features satisfying FC>1.5 or FC<0.67 were retained. Error (SD) was calculated from the SD of the relevant treated and untreated sample sets using appropriate rules for error propagation. All the hits from negative mode and only DG and TG species from positive mode are represented in this study. Lipid annotations and quantifications were performed following the guidelines of the Lipidomics Standard Initiative (LSI) (<https://lipidomics-standards-initiative.org/>).

**Targeted data analysis for lipid class comparisons**

We are building an in-house lipid database for common species including fatty acids, glycolipids, glycerophospholipids and sphingolipids, extracted from HeLa cells. This approach provides comprehensive information to investigate alteration trends in lipid species as groups in addition to investigating them individually. Unless stated otherwise, all lipid species are annotated by matching their MS/MS fragmentations. Raw ion counts of lipid species in our database were extracted from the initial dataset and plotted. Additionally, aggregated intensities of the signals from each species from the same class were used to plot and compare the ratio of the lipid classes. To create the database, Profinder batched targeted feature extraction was performed. LipidBlast [3] - in-silico tandem mass spectrometry database was employed to confirm the identity of major lipid species at specific retention times. Some species could not be identified by MS/MS analysis and were annotated by their accurate masses from LIPID MAPS and/or METLIN Metabolomics databases. We created several comma separated values (*.csv) files, which contain the identity and retention time of the lipid species from different classes. Identified features were extracted by choosing the “Batch Targeted Feature Extraction” option and using the *.csv files created above as the database. As the matching feature criteria, we selected the “mass and retention time required” option. H^+^, Na^+^ and NH_4_^+^ adducts were selected for positive mode and H^−^, CH_3_COO^−^ and HCOO^−^ adducts were selected for negative mode. Retention time (RT) span was set to 0.15 min and mass tolerance was set to 5 ppm. Automatically selected features were confirmed manually by their patterns of adducts and peak quality. We then exported (*.csv) files containing the identity, intensity and the retention time of each lipid species.

*.csv files from positive mode were used for extracting the DG and TG species while rest of the species were extracted from the negative mode analysis. Extracted intensities of individual lipid species were used to generate the bar plots of the lipid species and classes from all conditions. Error bars represent +/- standard deviation (n=6) for the lipid species and standard error of the mean (SEM) for the lipid classes.

**Supplementary References**

1. Sampaio, J.L., et al., Proc Natl Acad Sci U S A, **2011**. 108: p. 1903-7.

2. Cajka, T. and O. Fiehn, Metabolomics, **2016**. 12: p. 1-11.

3. Kind, T., et al., Nat Methods, **2013**. 10: p. 755-8.

4. Fahy, E. et al., [Nucleic Acids Res.](https://www.ncbi.nlm.nih.gov/pubmed/17584797?dopt=Abstract), **2007** W606-12

5. Smith, C.A., et al., [Ther Drug Monit.](https://www.ncbi.nlm.nih.gov/pubmed/16404815?dopt=Abstract), **2005**, 747-751
