# Supplementary material for: RNAi Transfection Results in Lipidome Changes
Source: Proteomics. 2019 Jun 13;19(13):1800298. doi: 10.1002/pmic.201800298 (PMC6617754; doi:10.1002/pmic.201800298)
Supplement: Supplementary file 2 — Supporting Information [file PMIC-19-na-s002.pdf]

PROTEOMICS

Supporting Information
for Proteomics
DOI 10.1002/pmic.201800298

Cagakan Özbalci, Elisabeth M. Storck and Ulrike S. Eggert

RNAi Transfection Results in Lipidome Changes

© 2019 The Authors. *Proteomics* published by WILEY-VCH Verlag GmbH & Co. KGaA, Weinheim.

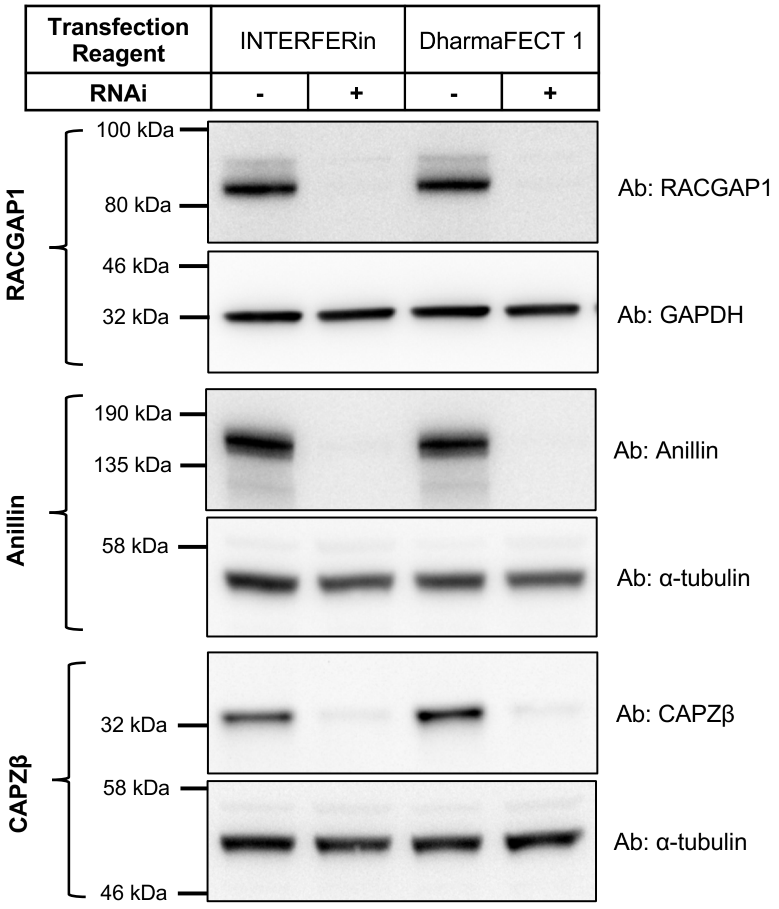
Transfection Reagent
INTERFERin
DharmaFECT 1
RNAi
-
+
-
+
RACGAP1
100 kDa
80 kDa
46 kDa
32 kDa
Ab: RACGAP1
Ab: GAPDH
Anillin
190 kDa
135 kDa
58 kDa
Ab: Anillin
Ab: α-tubulin
CAPZβ
32 kDa
58 kDa
46 kDa
Ab: CAPZβ
Ab: α-tubulin
