# Supplementary material for: RNAi Transfection Results in Lipidome Changes
Source: Proteomics. 2019 Jun 13;19(13):1800298. doi: 10.1002/pmic.201800298 (PMC6617754; doi:10.1002/pmic.201800298)
Supplement: Supplementary file 3 — Supporting Information [file PMIC-19-na-s003.pptx]

## Slide 1
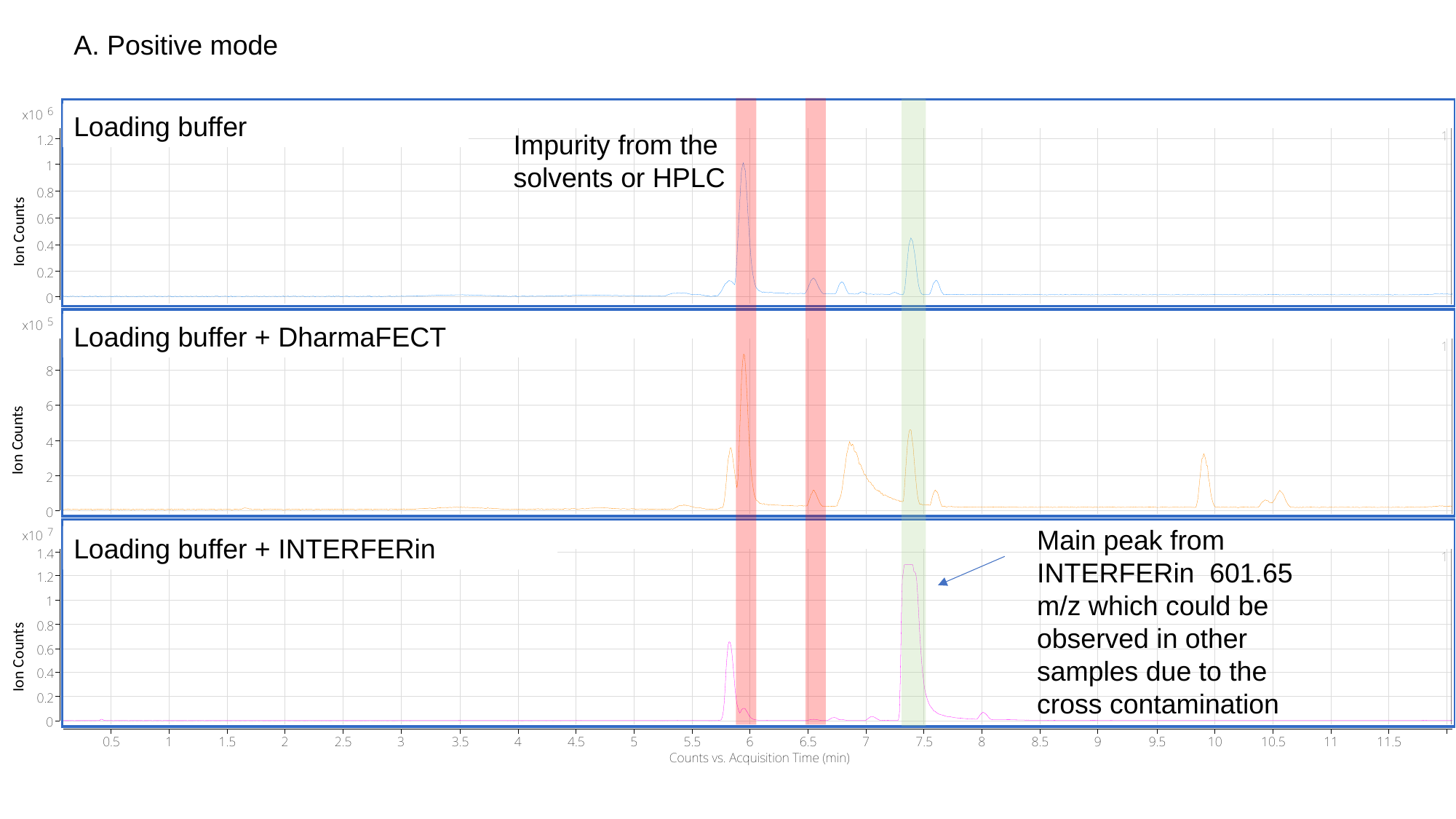

A. Positive mode
Loading buffer
Impurity from the solvents or HPLC
#
 Ion Counts
Loading buffer + DharmaFECT
 Ion Counts
Main peak from INTERFERin 601.65 m/z which could be observed in other samples due to the cross contamination
Loading buffer + INTERFERin
 Ion Counts

## Slide 2
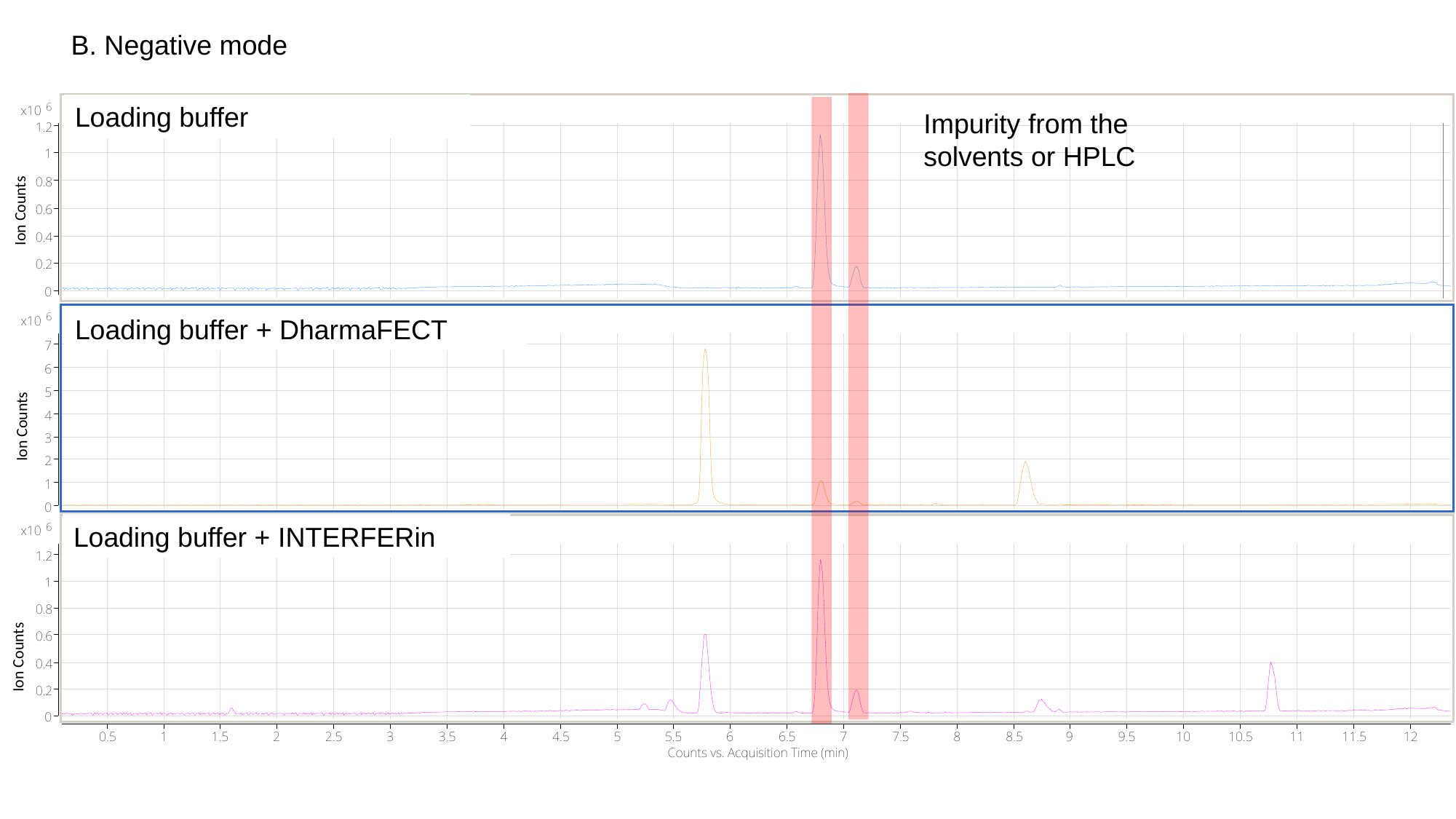

B. Negative mode
Loading buffer
Impurity from the solvents or HPLC
#
 Ion Counts
Loading buffer + DharmaFECT
 Ion Counts
Loading buffer + INTERFERin
 Ion Counts
