# Supplementary material for: RNAi Transfection Results in Lipidome Changes
Source: Proteomics. 2019 Jun 13;19(13):1800298. doi: 10.1002/pmic.201800298 (PMC6617754; doi:10.1002/pmic.201800298)
Supplement: Supplementary file 4 — Supporting Information [file PMIC-19-na-s004.docx]

**Cer d18:0_16:0**

Name: Cer[NDS] 34:0; Cer[NDS](d18:0/16:0); [M+Hac-H]-

Formula: C34H69NO3

MW: 539 ID#: 233 DB: cernds_hac_neg.msp

Comment: NA

Precursor m/z: 598.54106

**Cer d18:0_24:0**

Name: Cer[NDS] 42:0; Cer[NDS](d18:0/24:0); [M+Hac-H]-

Formula: C42H85NO3

MW: 651 ID#: 272 DB: cernds_hac_neg.msp

Comment: NA

Precursor m/z: 710.66626

**Cer d18:1_16:0**

Name: Cer[NS] 34:1; Cer[NS](d18:1/16:0); [M+Hac-H]-

Formula: C34H67NO3

MW: 537 ID#: 233 DB: cerns_hac_neg.msp

Comment: NA

Precursor m/z: 596.52541

**Cer d18:1_17:0**

Name: Cer[NS] 35:1; Cer[NS](d18:1/17:0); [M+Hac-H]-

Formula: C35H69NO3

MW: 551 ID#: 238 DB: cerns_hac_neg.msp

Comment: NA

Precursor m/z: 610.54106

**Cer d18:1_18:0**

Name: Cer[NS] 36:1; Cer[NS](d18:1/18:0); [M+Hac-H]-

Formula: C36H71NO3

MW: 565 ID#: 242 DB: cerns_hac_neg.msp

Comment: NA

Precursor m/z: 624.55671

**Cer[NS] 43:1**

Name: Cer[NS] 43:1; Cer[NS](d18:1/25:0); [M+Hac-H]-

Formula: C43H85NO3

MW: 663 ID#: 277 DB: cerns_hac_neg.msp

Comment: NA

Precursor m/z: 722.66626

**GM3 (d18:1/24:0)**

MW: 1263 ID#: 79294 DB: lipidblast-neg

Comment: Parent=1263.83054

5 m/z Values and Intensities:

290.08759 400.00 ion C11H16NO8- (290.08759)

648.62907 200.00 ion ceramide

810.68189 200.00 [M-H]-Cer-C6H10O5

972.73513 500.00 [M-H]-NeuAc-H

1263.83054 999.00 [M-H]-

**GM3 d18:1_22:0**

MW: 1235 ID#: 79293 DB: lipidblast-neg

Comment: Parent=1235.79924

5 m/z Values and Intensities:

290.08759 400.00 ion C11H16NO8- (290.08759)

620.59779 200.00 ion ceramide

782.65061 200.00 [M-H]-Cer-C6H10O5

944.70383 500.00 [M-H]-NeuAc-H

1235.79924 999.00 [M-H]-

**PC 18:1_22:4**

Name: PC 40:5; [M-Ac-H]

MW: 894 ID#: 3846 DB: pc-ac-neg.msp

Comment: Parent=894.62244

4 m/z Values and Intensities:

281.24790 100.00 FA sn1

331.26354 100.00 FA sn2

820.58566 999.00 [M-CH3]- (-15)

894.62244 100.00 [M+Ac-H]- (M+60-1)

**PE 16:0_16:1**

Name: PE 32:1; PE(16:0/16:1); [M-H]-

Formula: C37H72NO8P

MW: 689 ID#: 434 DB: pe_h_nega.msp

Precursor m/z: 688.49174

**PE 20:5_22:6**

Name: PE 42:11; PE(20:5/22:6); [M-H]-

Formula: C47H72NO8P

MW: 809 ID#: 1158 DB: pe_h_nega.msp

Precursor m/z: 808.49174

6 m/z Values and Intensities:

301.21662 999.00 sn1 FA

327.23226 999.00 sn2 FA

480.25167 50.00 [M-H]-sn2-H2O

498.26223 200.00 [M-H]-sn2

506.26731 50.00 [M-H]-sn1-H2O

524.27787200.00 [M-H]-sn1

**PE P-16:0_14:1**

Name: plasmenyl-PE 30:1; [M-H]-; PE(P-16:0/14:1)

MW: 644 ID#: 123228 DB: lipidblast-neg

Comment: Parent=644.46551

3 m/z Values and Intensities:

225.18534 999.00 sn2 FA

418.27235 50.00 [M-H]-sn2-H2O

436.28291 250.00 [M-H]-sn2 acyl chain

**PE P-16:0_12:0**

Name: plasmenyl-PE 28:0; [M-H]-; PE(P-16:0/12:0)

MW: 618 ID#: 123225 DB: lipidblast-neg

Comment: Parent=618.44988

3 m/z Values and Intensities:

199.16970 999.00 sn2 FA

418.27236 50.00 [M-H]-sn2-H2O

436.28292 250.00 [M-H]-sn2 acyl chain

**PE P-16:0_20:3**

Name: plasmenyl-PE 36:3; [M-H]-; PE(P-16:0/20:3)

MW: 724 ID#: 123265 DB: lipidblast-neg

Comment: Parent=724.52813

3 m/z Values and Intensities:

305.24790 999.00 sn2 FA

418.27241 50.00 [M-H]-sn2-H2O

436.28297 250.00 [M-H]-sn2 acyl chain

**PG 18:1_18:1**

Name: PG 36:2; [M-H]-; GPGro(18:1/18:1)

MW: 773 ID#: 113988 DB: lipidblast-neg

Comment: Parent=773.53326 Mz_

4 m/z Values and Intensities:

281.24790 999.00 sn1 FA || sn2 FA

417.24076 200.00 [M-H]-sn1-C3H8O3 || [M-H]-sn2-C3H8O3

491.27754 200.00 [M-H]-sn1-H2O || [M-H]-sn2-H2O

509.28810 200.00 [M-H]-sn1 || [M-H]-sn2

**PG 18:1_22:6**

MW: 819 ID#: 114028 DB: lipidblast-neg

Comment: Parent=819.51763

8 m/z Values and Intensities:

281.24790 999.00 sn1 FA

327.23226 999.00 sn2 FA

417.24077 200.00 [M-H]-sn2-C3H8O3

463.22513 200.00 [M-H]-sn1-C3H8O3

491.27755 200.00 [M-H]-sn2-H2O

509.28811 200.00 [M-H]-sn2

537.26191 200.00 [M-H]-sn1-H2O

555.27247 200.00 [M-H]-sn1

**PG 16:1_22:6**

MW: 791 ID#: 113584 DB: lipidblast-neg

Comment: Parent=791.48632

8 m/z Values and Intensities:

253.21662 999.00 sn1 FA

327.23226 999.00 sn2 FA

389.20946 200.00 [M-H]-sn2-C3H8O3

463.22510 200.00 [M-H]-sn1-C3H8O3

463.24624 200.00 [M-H]-sn2-H2O

481.25680 200.00 [M-H]-sn2

537.26188 200.00 [M-H]-sn1-H2O

555.27244 200.00 [M-H]-sn1

**PG 22:5_22:6**

MW: 867 ID#: 116840 DB: lipidblast-neg

Comment: Parent=867.51763 Mz_

8 m/z Values and Intensities:

327.23226 999.00 sn2 FA

329.24790 999.00 sn1 FA

463.22513 200.00 [M-H]-sn1-C3H8O3

465.24077 200.00 [M-H]-sn2-C3H8O3

537.26191 200.00 [M-H]-sn1-H2O

539.27755 200.00 [M-H]-sn2-H2O

555.27247 200.00 [M-H]-sn1

557.28811 200.00 [M-H]-sn2

**PG 22:6_22:6**

MW: 865 ID#: 116988 DB: lipidblast-neg

Comment: Parent=865.50195

4 m/z Values and Intensities:

327.23226 999.00 sn1 FA || sn2 FA

463.22509 200.00 [M-H]-sn1-C3H8O3 || [M-H]-sn2-C3H8O3

537.26187 200.00 [M-H]-sn1-H2O || [M-H]-sn2-H2O

555.27243 200.00 [M-H]-sn1 || [M-H]-sn2

**TG 48:1**

Name: TG 48:1; [M+NH4]+; TG(14:0/16:0/18:1)

MW: 822 ID#: 75692 DB: lipidblast-pos

Comment: Parent=822.75461

4 m/z Values and Intensities:

523.47234 999.00 [M+NH4]-sn3-18

549.48798 999.00 [M+NH4]-sn2-18

577.51926 999.00 [M+NH4]-sn1-18

805.75187 199.80 [M+NH4]-17

**TG 54:6**

Name: TG 54:6; [M+NH4]+; TG(16:0/16:1/22:5)

MW: 896 ID#: 75752 DB: lipidblast-pos

Comment: Parent=896.77025 Mz_exact=896.77025 ; TG 54:6; [M+NH4]+; TG(16:0/16:1/22:5); C57H98O6

4 largest peaks:

549.48798 999.00 | 623.50362 999.00 | 625.51926 999.00 | 879.76751 199.80 |

4 m/z Values and Intensities:

549.48798 999.00 [M+NH4]-sn3-18

623.50362 999.00 [M+NH4]-sn1-18

625.51926 999.00 [M+NH4]-sn2-18

879.76751 199.80 [M+NH4]-17

**TG 56:6**

Name: TG 56:6; [M+NH4]+; TG(16:0/18:1/22:5)

MW: 924 ID#: 75853 DB: lipidblast-pos

Comment: Parent=924.80153

4 m/z Values and Intensities:

577.51926 999.00 [M+NH4]-sn3-18

625.51926 999.00 [M+NH4]-sn2-18

651.53490 999.00 [M+NH4]-sn1-18

907.79879 199.80 [M+NH4]-17

**TG 58:8**

Name: TG 58:8; [M+NH4]+; TG(18:1/18:1/22:6)

MW: 948 ID#: 77188 DB: lipidblast-pos

Comment: Parent=948.80153 Mz_exact=948.80153 ; TG 58:8; [M+NH4]+; TG(18:1/18:1/22:6); C61H102O6

3 largest peaks:

603.53490 999.00 | 649.51926 999.00 | 931.79879 199.80 |

3 m/z Values and Intensities:

603.53490 999.00 [M+NH4]-sn3-18

649.51926 999.00 [M+NH4]-sn1-18 || [M+NH4]-sn
