# Supplementary material for: RNAi Transfection Results in Lipidome Changes
Source: Proteomics. 2019 Jun 13;19(13):1800298. doi: 10.1002/pmic.201800298 (PMC6617754; doi:10.1002/pmic.201800298)
Supplement: Supplementary file 5 — Supporting Information [file PMIC-19-na-s005.pptx]

## Slide 1
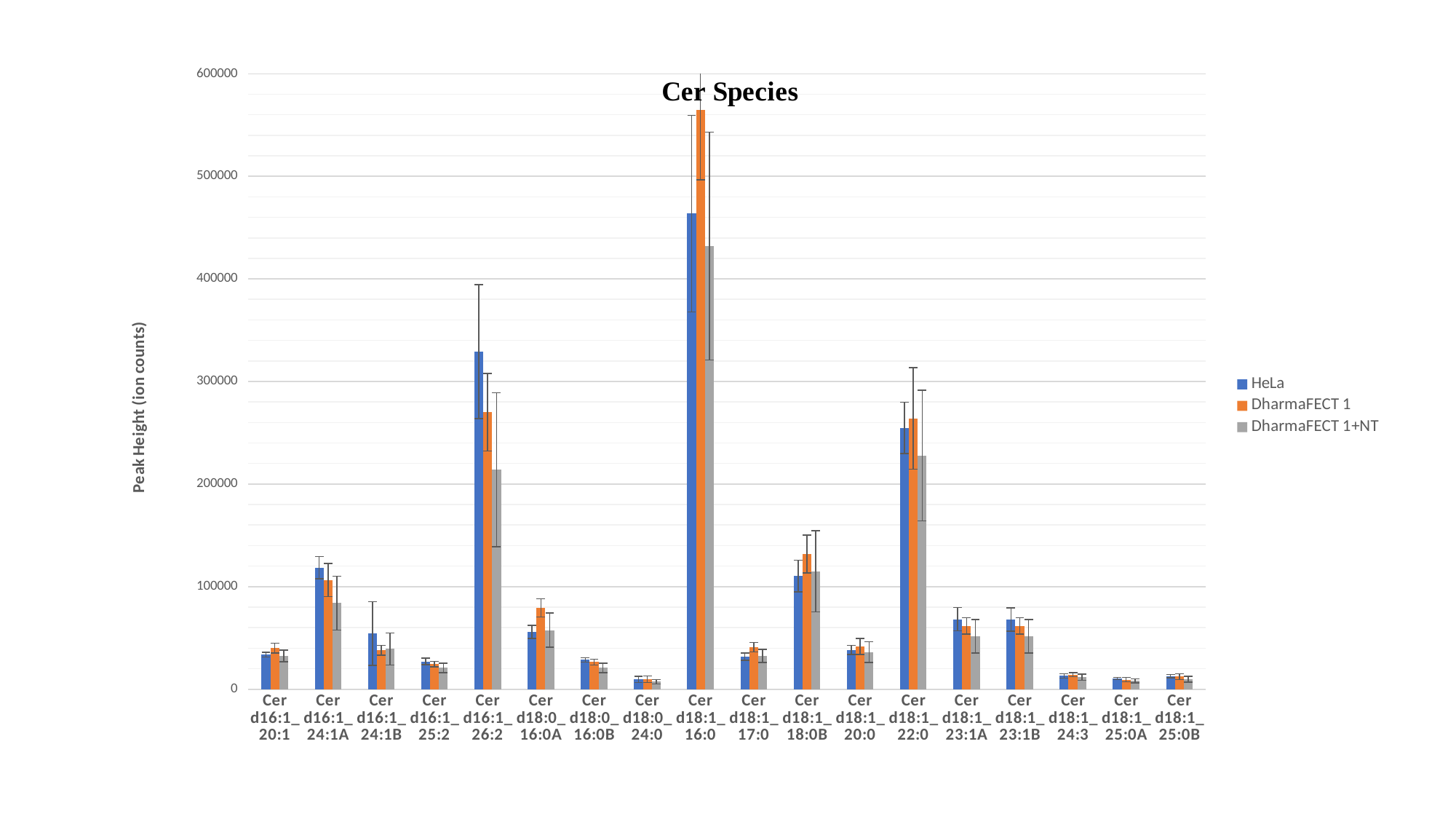

### Chart
| Category | HeLa | DharmaFECT 1 | DharmaFECT 1+NT |
|---|---|---|---|
| Cer d16:1_20:1 | 33582.66919962564 | 40154.81681315106 | 32411.3987833659 |
| Cer d16:1_24:1A | 118471.6136963296 | 106336.2538597263 | 83985.89724254927 |
| Cer d16:1_24:1B | 54282.54076183121 | 37890.67028841518 | 39204.6979827349 |
| Cer d16:1_25:2 | 26998.59044254227 | 24471.2755052456 | 20752.41108423658 |
| Cer d16:1_26:2 | 329019.3142631615 | 270079.1844741717 | 214048.6118350355 |
| Cer d18:0_16:0A | 55832.77953338623 | 79222.30781046551 | 57658.93187459312 |
| Cer d18:0_16:0B | 28553.20959472657 | 26475.27313232424 | 20914.70098876955 |
| Cer d18:0_24:0 | 9614.126210530592 | 9816.449020385748 | 7331.045939127605 |
| Cer d18:1_16:0 | 463613.9754231772 | 564590.2922363282 | 431973.044026693 |
| Cer d18:1_17:0 | 31841.40574977915 | 41027.89068007945 | 32637.46806964116 |
| Cer d18:1_18:0B | 110328.8953959147 | 131761.1591288248 | 115013.4641952514 |
| Cer d18:1_20:0 | 38292.90401361543 | 41757.19743866998 | 36167.1843921779 |
| Cer d18:1_22:0 | 254729.3001330732 | 263852.3190820874 | 227779.3496516861 |
| Cer d18:1_23:1A | 68142.88953249028 | 61774.76426319731 | 51634.23664280016 |
| Cer d18:1_23:1B | 67952.88133834393 | 61767.411297825 | 51493.7772614693 |
| Cer d18:1_24:3 | 13062.41625976562 | 14184.96339925128 | 11607.16057840983 |
| Cer d18:1_25:0A | 10394.60738118489 | 9276.05379231772 | 8123.859008789065 |
| Cer d18:1_25:0B | 12799.8856608073 | 12246.78212483724 | 9867.74997456868 |

## Slide 2
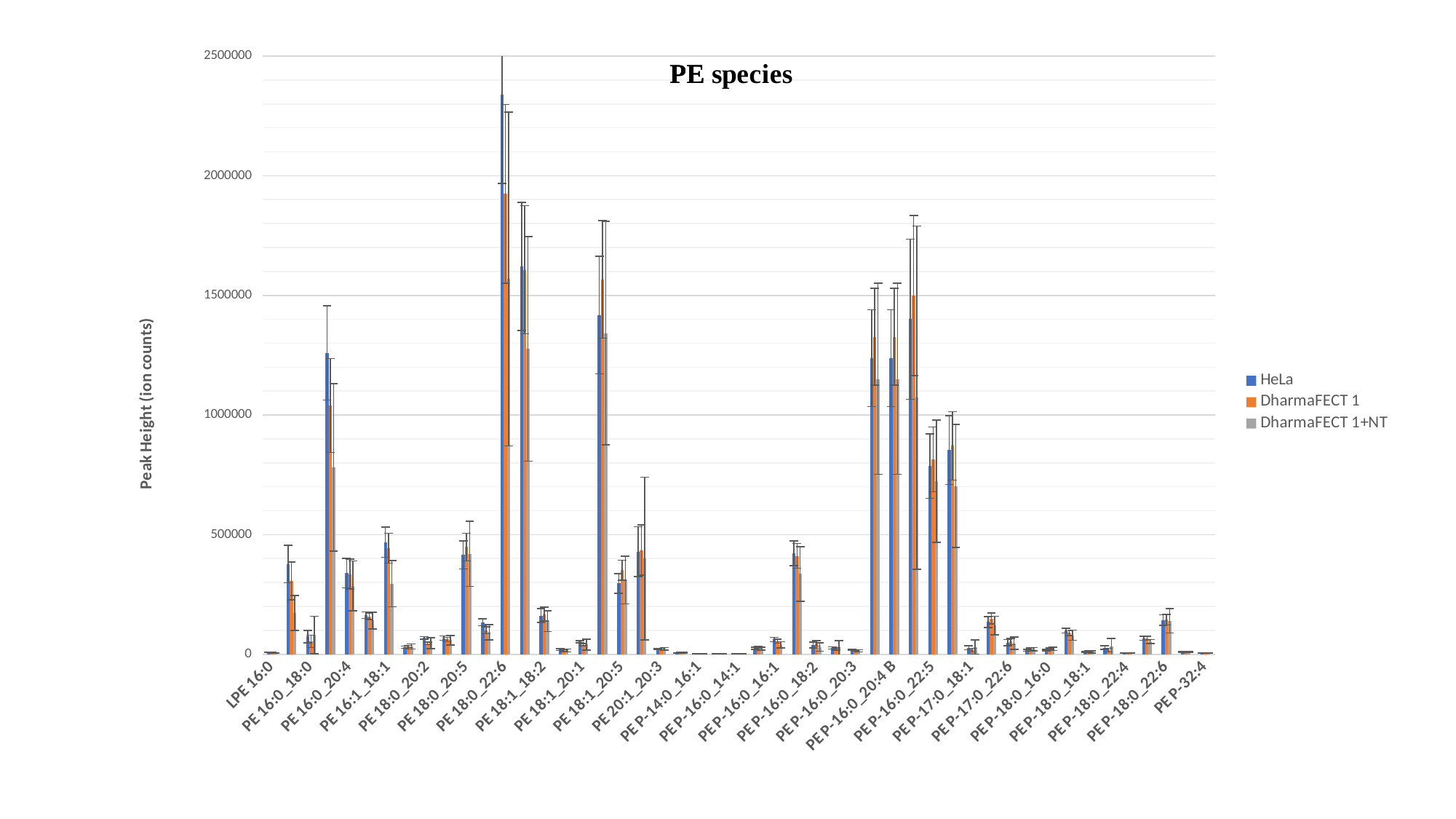

### Chart
| Category | HeLa | DharmaFECT 1 | DharmaFECT 1+NT |
|---|---|---|---|
| LPE 16:0 | 9476.513366699217 | 7451.659192403158 | 5994.799883524577 |
| PE 16:0_16:1 | 377540.884765625 | 306632.3958333333 | 172886.4514973957 |
| PE 16:0_18:0 | 73890.71997070307 | 54300.19771321616 | 80427.84678141275 |
| PE 16:0_18:1 | 1259638.31445312 | 1039560.57421875 | 781496.7112630216 |
| PE 16:0_20:4 | 339028.861246745 | 335373.3999023438 | 286222.8174235029 |
| PE 16:0_20:5 | 163843.3623860677 | 158369.5244140628 | 140740.0399576823 |
| PE 16:1_18:1 | 468439.7640740546 | 443367.6083190658 | 294626.4718523154 |
| PE 17:0_20:4 | 29993.11979166666 | 32287.7703450521 | 32685.59383138022 |
| PE 18:0_20:2 | 68630.39331054689 | 46113.56042480469 | 46455.12532552082 |
| PE 18:0_20:3 | 67428.93749999999 | 62132.67692057291 | 59146.70092773437 |
| PE 18:0_20:5 | 415330.6963021224 | 448206.9046148136 | 419567.4271946533 |
| PE 18:0_22:5 | 133880.0861332223 | 100865.8531479 | 92230.23248586306 |
| PE 18:0_22:6 | 2340352.14757077 | 1924776.35820785 | 1568126.2349891 |
| PE 18:1_18:1 | 1620856.64874438 | 1607131.61141937 | 1276019.79670538 |
| PE 18:1_18:2 | 161453.6598865923 | 167483.0942586355 | 138471.2211991026 |
| PE 18:1_20:0 | 20220.00813802086 | 15647.67187500003 | 16150.82165527345 |
| PE 18:1_20:1 | 52927.12825520834 | 40127.09122721355 | 40341.03149414062 |
| PE 18:1_20:3 | 1417413.90890702 | 1567488.67115398 | 1342401.10112372 |
| PE 18:1_20:5 | 295873.3190554945 | 351650.4320453372 | 310318.7208938927 |
| PE 18:1_22:6 | 428888.6576011612 | 435598.3657118885 | 399630.9325475805 |
| PE 20:1_20:3 | 21129.56380208334 | 21880.67643229167 | 22111.62760416667 |
| PE 20:5_22:6 | 5574.104593912762 | 8775.109115600584 | 7439.842768351234 |
| PE P-14:0_16:1 | 2917.07214609782 | 2869.840548197428 | 2442.64096069336 |
| PE P-16:0_12:0 | 1997.709869384767 | 2388.526662190754 | 2258.588694254557 |
| PE P-16:0_14:1 | 2554.644083658855 | 2815.953285217285 | 2283.846649169921 |
| PE P-16:0_16:0 | 24758.86494954427 | 30422.6288655599 | 23299.9958190918 |
| PE P-16:0_16:1 | 61830.00483194988 | 55223.49800618491 | 39570.91696166993 |
| PE P-16:0_18:1 | 422053.7426324685 | 411415.8242500388 | 335552.70973273 |
| PE P-16:0_18:2 | 38661.12679036459 | 45542.3203125 | 30558.4666341146 |
| PE P-16:0_20:3 | 26190.14827473958 | 22014.5234375 | 27037.70768229167 |
| PE P-16:0_20:3 | 18470.9020182292 | 16120.46748860679 | 14911.11301676434 |
| PE P-16:0_20:4 A | 1237100.76009115 | 1326994.73046875 | 1150807.03320313 |
| PE P-16:0_20:4 B | 1237100.76009115 | 1326994.73046875 | 1150807.03320313 |
| PE P-16:0_20:5 | 1400554.30338542 | 1499119.88671875 | 1072037.68033854 |
| PE P-16:0_22:5 | 786528.1897786458 | 814932.8395182289 | 723352.0105794271 |
| PE P-16:0_22:6 | 853361.2651936533 | 871056.440730371 | 702933.5381959976 |
| PE P-17:0_18:1 | 27729.21248372397 | 17439.43017578127 | 30474.15954589844 |
| PE P-17:0_20:4 | 134722.3661499025 | 149738.0814208985 | 119702.6072998048 |
| PE P-17:0_22:6 | 48989.96126302084 | 53599.1385091146 | 45980.40958658857 |
| PE P-18:0_16:0 | 16770.23700968425 | 22670.37223307293 | 21761.39097086589 |
| PE P-18:0_16:0 | 17364.12697347005 | 23153.53729248048 | 22638.23634847005 |
| PE P-18:0_18:1 | 98570.66975911464 | 88756.6289876303 | 79310.3174641928 |
| PE P-18:0_18:1 | 8925.12402343751 | 12878.11710611981 | 10068.6360880534 |
| PE P-18:0_20:3 | 27765.71899414062 | 17468.35896809895 | 31692.53564453126 |
| PE P-18:0_22:4 | 5666.680257161458 | 4862.995279947917 | 5286.622884114583 |
| PE P-18:0_22:5 | 67310.79459635417 | 67224.61067708333 | 53505.63736979165 |
| PE P-18:0_22:6 | 143316.1376557297 | 144502.4932057348 | 139811.717691932 |
| PE P-20:0_22:6 | 10146.35003662109 | 10718.12522888184 | 9147.44623819987 |
| PE P-32:4 | 5439.693949381513 | 4894.351501464848 | 3982.170979817711 |

## Slide 3
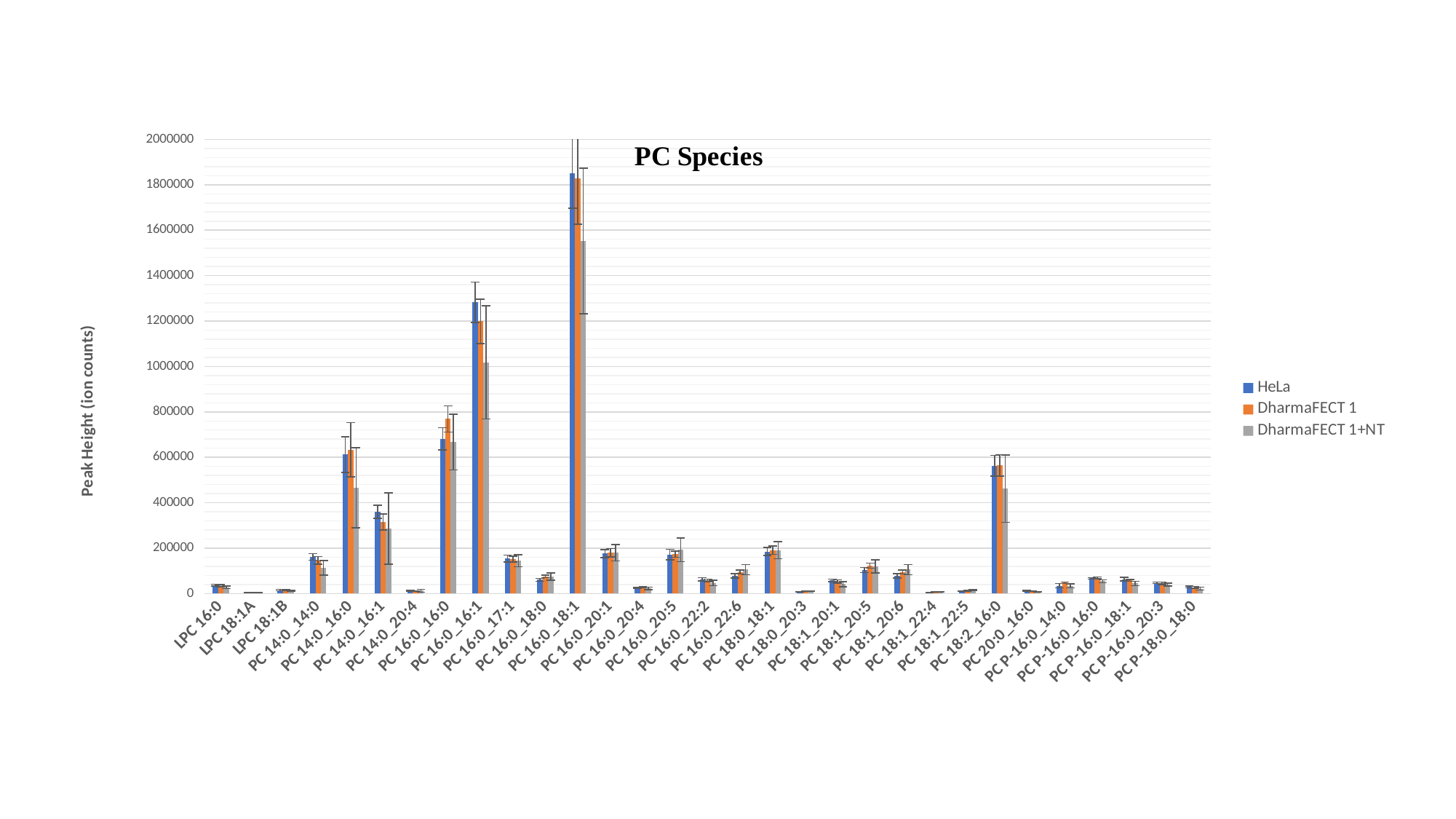

### Chart
| Category | HeLa | DharmaFECT 1 | DharmaFECT 1+NT |
|---|---|---|---|
| LPC 16:0 | 37812.873819987 | 34872.97528076169 | 27410.15004475913 |
| LPC 18:1A | 4085.196329752605 | 4444.038045247396 | 3419.647735595705 |
| LPC 18:1B | 15624.861521403 | 15997.22977193197 | 11784.18659210206 |
| PC 14:0_14:0 | 161549.00825 | 145973.2681333333 | 113346.002405 |
| PC 14:0_16:0 | 612002.0102666666 | 633363.1787166664 | 465724.5937333334 |
| PC 14:0_16:1 | 360461.9547833333 | 315195.7837333334 | 286381.3805333334 |
| PC 14:0_20:4 | 11728.604247 | 9751.787840500003 | 12617.49772133333 |
| PC 16:0_16:0 | 681470.3707499997 | 769099.4824333334 | 667258.6796833335 |
| PC 16:0_16:1 | 1282900.04066667 | 1198685.20566667 | 1017846.84478333 |
| PC 16:0_17:1 | 154331.8613166667 | 152058.4563833333 | 144845.0410166667 |
| PC 16:0_18:0 | 61053.04134 | 76427.48177333333 | 75054.98274666663 |
| PC 16:0_18:1 | 1849168.83216667 | 1828874.4885 | 1553163.57683333 |
| PC 16:0_20:1 | 176197.1736666666 | 179723.194 | 179888.2765166667 |
| PC 16:0_20:4 | 25695.49039833333 | 28531.83919333333 | 22500.60270333333 |
| PC 16:0_20:5 | 171858.3347833333 | 173636.6603166667 | 192923.5296333333 |
| PC 16:0_22:2 | 63020.55118833332 | 58239.79998833331 | 46739.46531166664 |
| PC 16:0_22:6 | 78021.931965 | 95144.75489666664 | 105736.4479183333 |
| PC 18:0_18:1 | 185644.2367666667 | 191846.9767333333 | 191115.43795 |
| PC 18:0_20:3 | 8610.539916833333 | 10653.47859666666 | 11064.3802895 |
| PC 18:1_20:1 | 57867.512165 | 53232.05932333333 | 41792.45166166665 |
| PC 18:1_20:5 | 104162.873395 | 123293.5963333333 | 119166.9972333333 |
| PC 18:1_20:6 | 78021.931965 | 95144.75489666664 | 105865.4309916667 |
| PC 18:1_22:4 | 4423.505778166667 | 7149.130899833332 | 7770.375691833334 |
| PC 18:1_22:5 | 10831.00423 | 12446.39298333333 | 14877.54044666667 |
| PC 18:2_16:0 | 562633.2740999996 | 564946.7792999996 | 461808.3711 |
| PC 20:0_16:0 | 11726.38606916667 | 10528.15095983333 | 8134.439941333332 |
| PC P-16:0_14:0 | 34688.46407 | 48327.02897 | 34202.20662333332 |
| PC P-16:0_16:0 | 67604.8925816666 | 69499.09993666664 | 54304.57747333332 |
| PC P-16:0_18:1 | 63681.51135 | 58417.76039666665 | 44969.93562833333 |
| PC P-16:0_20:3 | 47360.22440499999 | 45720.36470666665 | 40358.75298833333 |
| PC P-18:0_18:0 | 29298.36979333333 | 27850.193605 | 22097.71695833332 |

## Slide 4
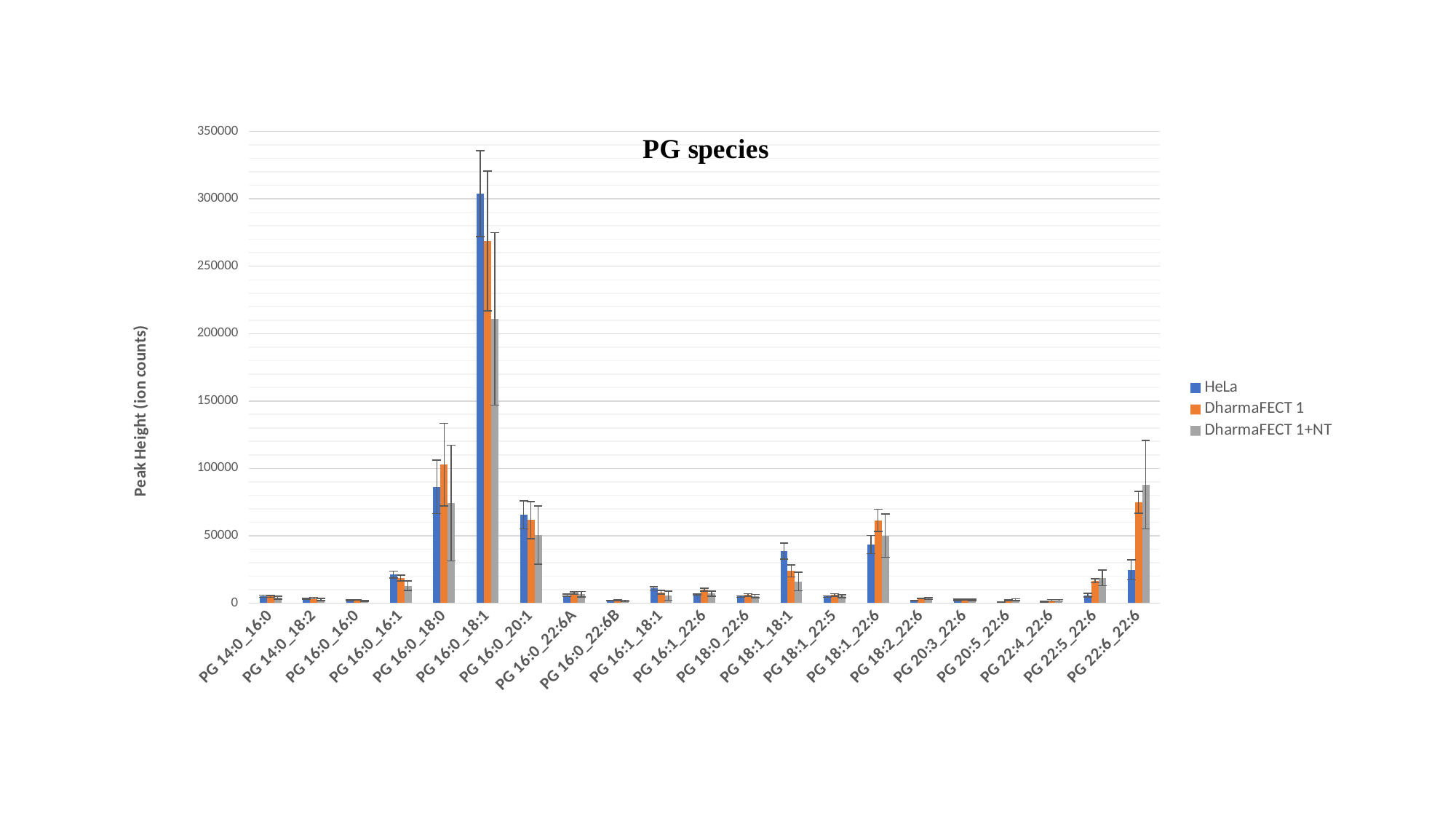

### Chart
| Category | HeLa | DharmaFECT 1 | DharmaFECT 1+NT |
|---|---|---|---|
| PG 14:0_16:0 | 5144.43304189046 | 4907.015141805013 | 3889.302541097006 |
| PG 14:0_18:2 | 3273.066551208497 | 3636.221585591636 | 2589.58401743571 |
| PG 16:0_16:0 | 2232.661544799803 | 2170.9508005778 | 1620.488255818683 |
| PG 16:0_16:1 | 21056.44326019287 | 18638.98043823242 | 12874.23249562583 |
| PG 16:0_18:0 | 86258.55744732301 | 102706.4297939138 | 74252.6896385214 |
| PG 16:0_18:1 | 303882.3018096455 | 268784.9323131878 | 211022.3489938897 |
| PG 16:0_20:1 | 65447.34448242191 | 61601.70385742191 | 50450.47615559898 |
| PG 16:0_22:6A | 5797.813771565755 | 7394.898091634112 | 6594.703226725261 |
| PG 16:0_22:6B | 1904.80458577474 | 2083.141265869143 | 1556.054178873698 |
| PG 16:1_18:1 | 10928.34643554687 | 7996.74057006836 | 5422.50609842936 |
| PG 16:1_22:6 | 6024.81266276042 | 9978.220901489249 | 7010.748372395831 |
| PG 18:0_22:6 | 4912.624348958335 | 5991.641031901043 | 5110.728434244791 |
| PG 18:1_18:1 | 38454.65917968749 | 23860.51651000978 | 16042.68909708662 |
| PG 18:1_22:5 | 4823.337677001954 | 5958.456115722659 | 4932.059672037761 |
| PG 18:1_22:6 | 43363.5491205851 | 61368.16571807861 | 50040.1285909017 |
| PG 18:2_22:6 | 1939.091224670411 | 3503.691057840984 | 3264.042673746746 |
| PG 20:3_22:6 | 2427.847544352215 | 2601.191792805992 | 2244.571848551434 |
| PG 20:5_22:6 | 952.1088765462247 | 2047.03415934245 | 2402.324259440105 |
| PG 22:4_22:6 | 1073.306546529134 | 2030.637959798177 | 2008.68559773763 |
| PG 22:5_22:6 | 5743.928064982097 | 16583.45196533205 | 18688.35587565105 |
| PG 22:6_22:6 | 24742.02728271486 | 74809.82725016274 | 87771.39326985687 |

## Slide 5
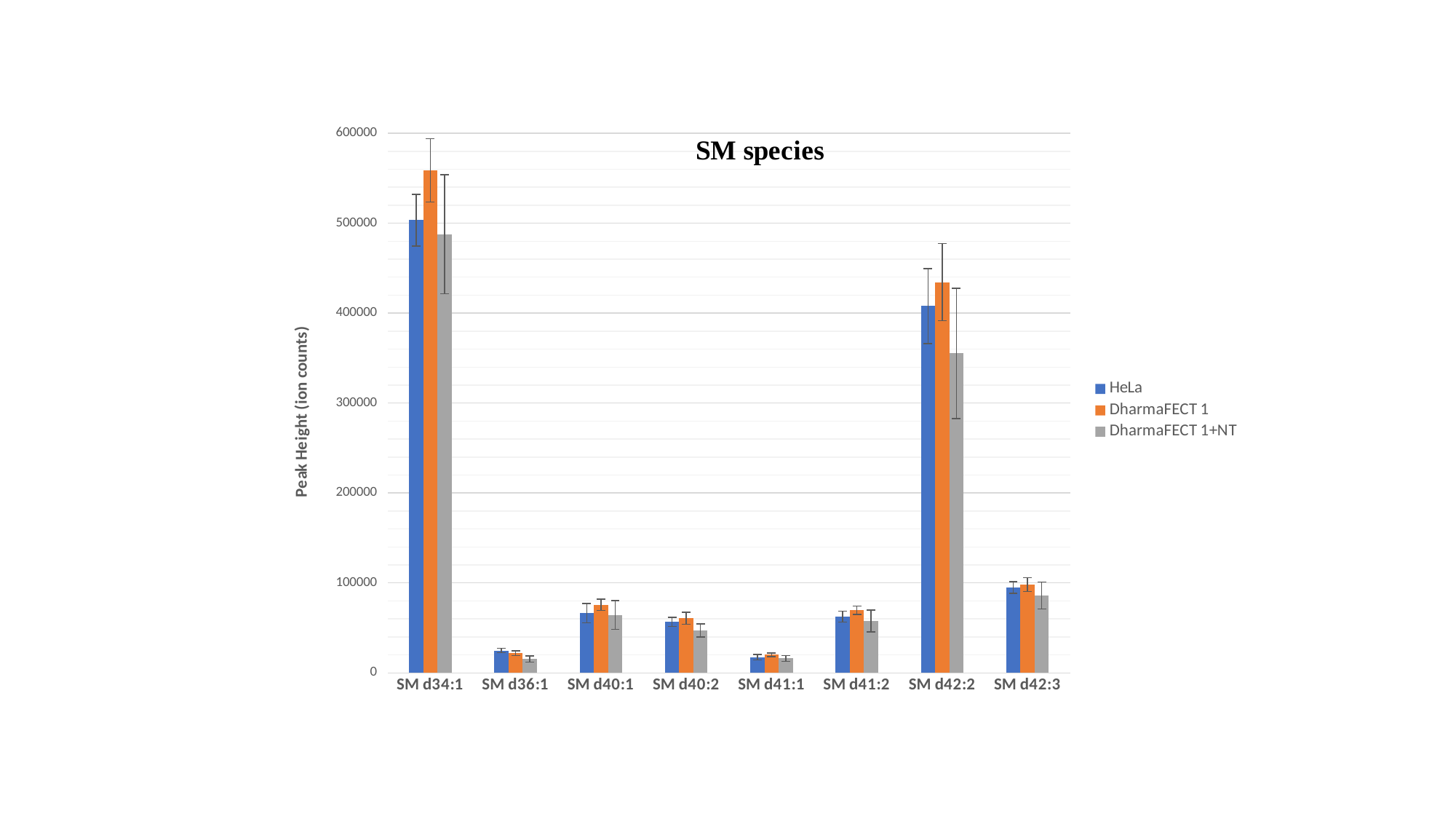

### Chart
| Category | HeLa | DharmaFECT 1 | DharmaFECT 1+NT |
|---|---|---|---|
| SM d34:1 | 503466.8441724401 | 558709.7927751177 | 487830.6767286338 |
| SM d36:1 | 24786.09629313152 | 21756.17198181152 | 15431.54650624593 |
| SM d40:1 | 66366.1005859375 | 75593.41259765619 | 64381.05078125 |
| SM d40:2 | 56687.57529245971 | 60848.04113089634 | 47184.63194067264 |
| SM d41:1 | 17414.50154622396 | 20030.67594401042 | 16065.94213867188 |
| SM d41:2 | 62371.03869047418 | 69616.97597311181 | 57572.40594496601 |
| SM d42:2 | 407834.9565343231 | 434475.8056534956 | 355301.0240211126 |
| SM d42:3 | 95042.50319417329 | 98016.96579996744 | 86006.29133097331 |

## Slide 6
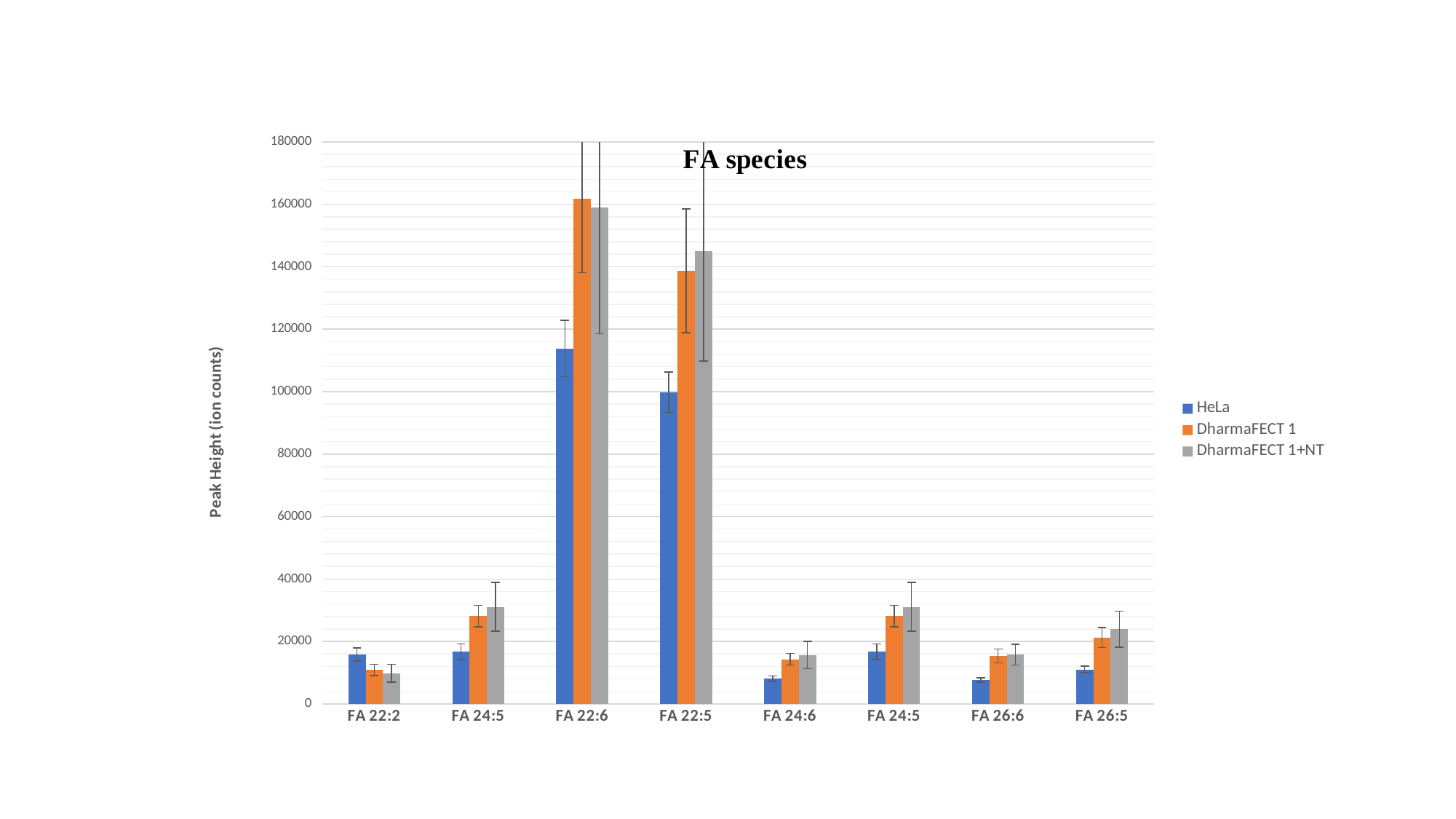

#
### Chart
| Category | HeLa | DharmaFECT 1 | DharmaFECT 1+NT |
|---|---|---|---|
| FA 22:2 | 15822.55624706612 | 10879.52563476562 | 9834.746487645516 |
| FA 24:5 | 16666.09602167176 | 28114.59383247147 | 31081.30271149958 |
| FA 22:6 | 113832.883292559 | 161786.9966204843 | 158953.9735331622 |
| FA 22:5 | 99825.54900180077 | 138705.0797156032 | 145042.5009519886 |
| FA 24:6 | 8084.998163573975 | 14287.74234879112 | 15684.31105610862 |
| FA 24:5 | 16665.4888072784 | 28117.36871341558 | 31080.57066818362 |
| FA 26:6 | 7639.892733058894 | 15336.93512732557 | 15764.74370067717 |
| FA 26:5 | 11027.96342817656 | 21271.23772144108 | 23917.87080514227 |

## Slide 7
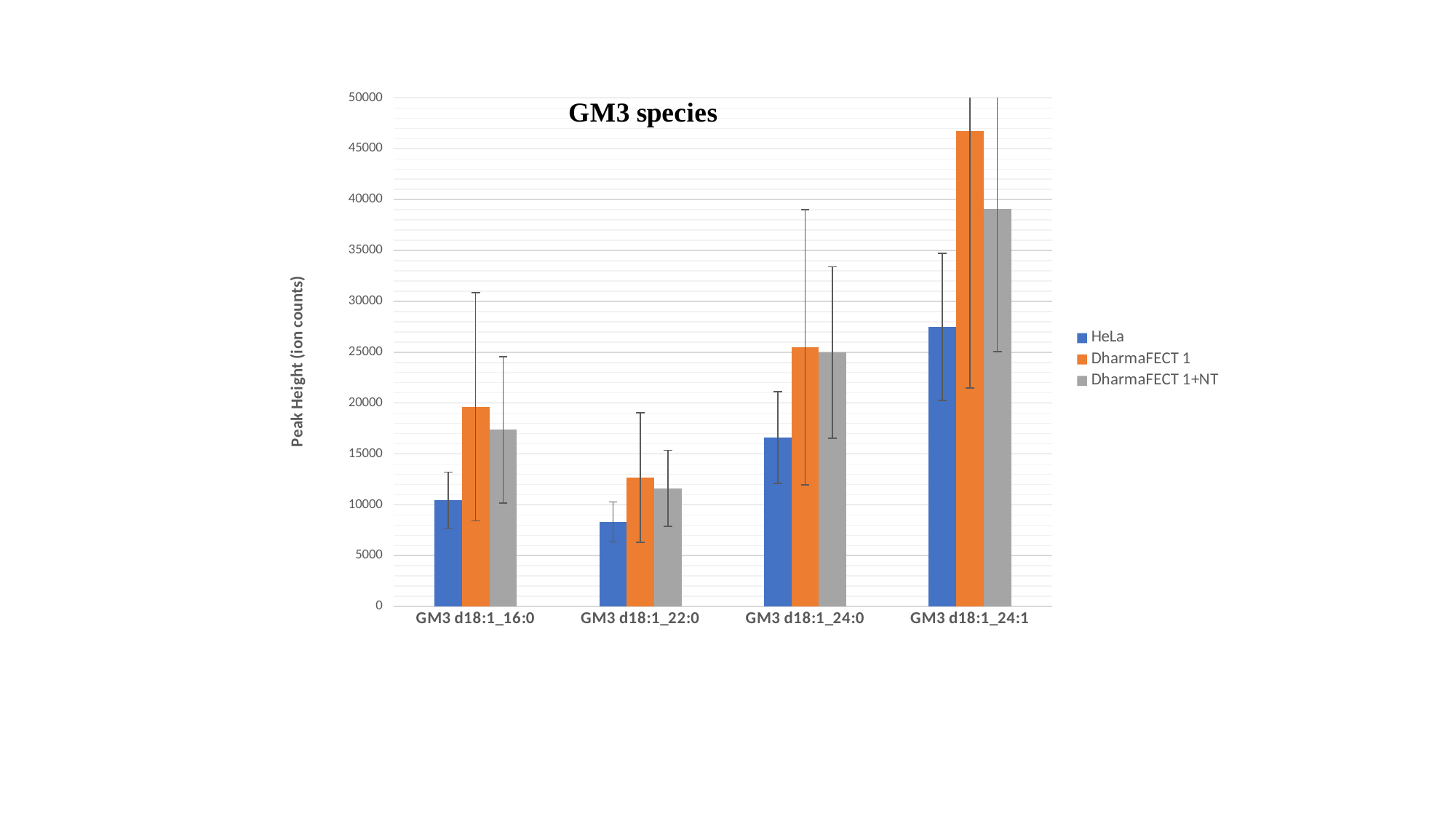

### Chart
| Category | HeLa | DharmaFECT 1 | DharmaFECT 1+NT |
|---|---|---|---|
| GM3 d18:1_16:0 | 10456.00112676847 | 19632.89906714169 | 17363.42860898091 |
| GM3 d18:1_22:0 | 8319.976651778941 | 12672.53197959107 | 11629.42264721397 |
| GM3 d18:1_24:0 | 16617.40447615272 | 25475.60577243142 | 24957.7652276837 |
| GM3 d18:1_24:1 | 27478.85674521421 | 46777.22904933884 | 39077.39367680057 |

## Slide 8
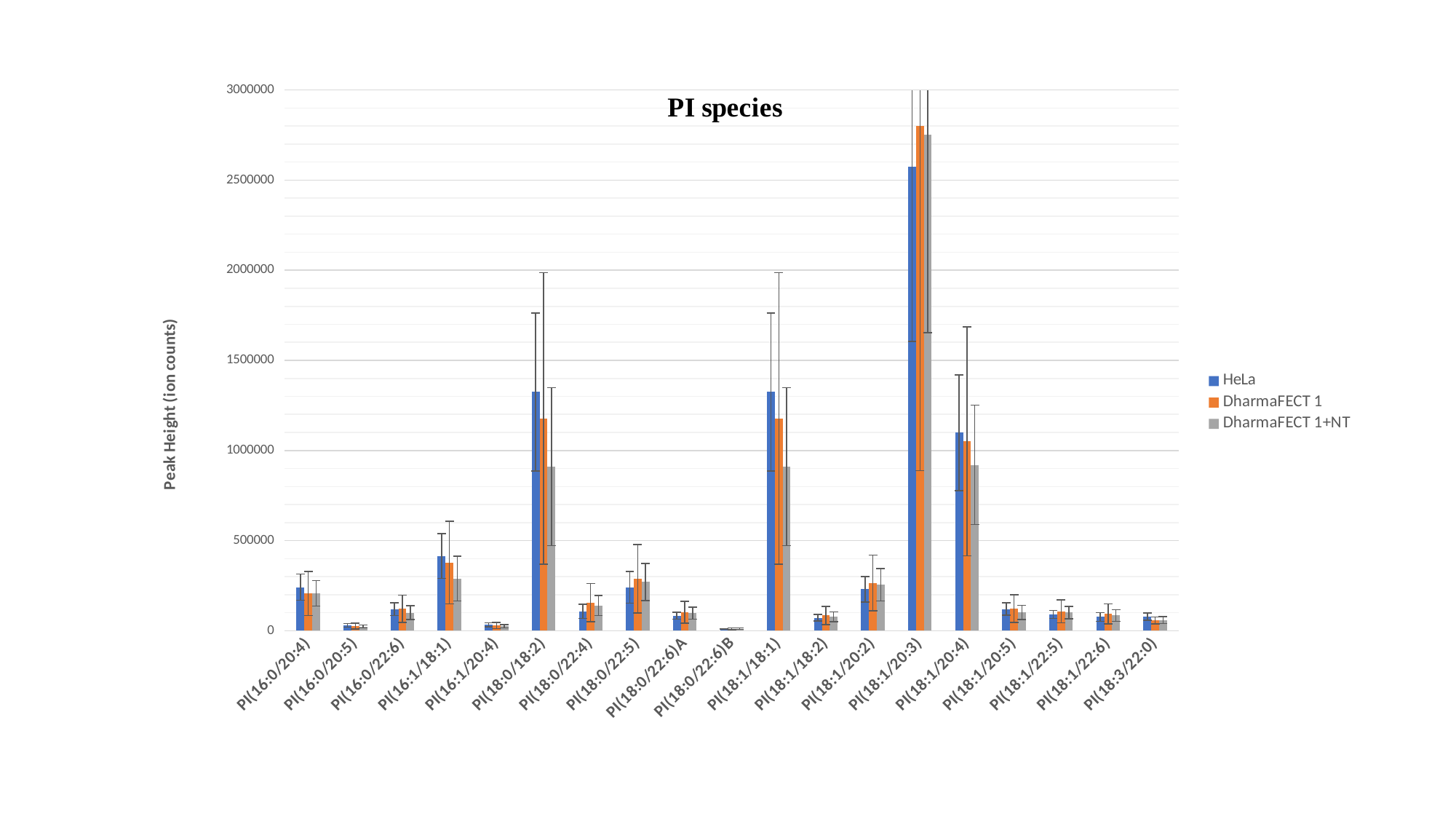

### Chart
| Category | HeLa | DharmaFECT 1 | DharmaFECT 1+NT |
|---|---|---|---|
| PI(16:0/20:4) | 241795.2272899475 | 206217.2904783262 | 207276.8089073201 |
| PI(16:0/20:5) | 30281.35184733074 | 25559.8260904948 | 22997.02126057943 |
| PI(16:0/22:6) | 119503.9985148112 | 121747.824564616 | 99974.8644409179 |
| PI(16:1/18:1) | 415071.2738035347 | 378072.0287331347 | 289077.0764946635 |
| PI(16:1/20:4) | 32554.3044290673 | 28015.83258738121 | 25198.63502653355 |
| PI(18:0/18:2) | 1324814.06463961 | 1177998.70012679 | 909899.3167409503 |
| PI(18:0/22:4) | 107745.4314121603 | 155503.7955790207 | 138999.6487308751 |
| PI(18:0/22:5) | 240445.1143265101 | 287704.1068966144 | 270034.7621150206 |
| PI(18:0/22:6)A | 83464.0565185547 | 102802.172526042 | 97399.11657714858 |
| PI(18:0/22:6)B | 9261.576904296888 | 11333.22515869142 | 11580.08622233073 |
| PI(18:1/18:1) | 1324796.37970103 | 1178060.72054434 | 909858.4887406073 |
| PI(18:1/18:2) | 71915.68520100911 | 84433.22949218754 | 76665.31990559898 |
| PI(18:1/20:2) | 230336.6892604595 | 264334.9281324165 | 254591.7647067175 |
| PI(18:1/20:3) | 2573701.3873673 | 2801017.46422279 | 2752494.83076212 |
| PI(18:1/20:4) | 1099008.82189863 | 1050169.90646284 | 919851.0983385156 |
| PI(18:1/20:5) | 120418.0044293676 | 123574.1532308251 | 102063.6075898042 |
| PI(18:1/22:5) | 90387.93497922644 | 108285.8988998625 | 100435.2394218483 |
| PI(18:1/22:6) | 76932.6941931805 | 93271.3838004917 | 84344.12329281475 |
| PI(18:3/22:0) | 78437.63696917637 | 57195.96977116343 | 59147.38368646591 |

## Slide 9
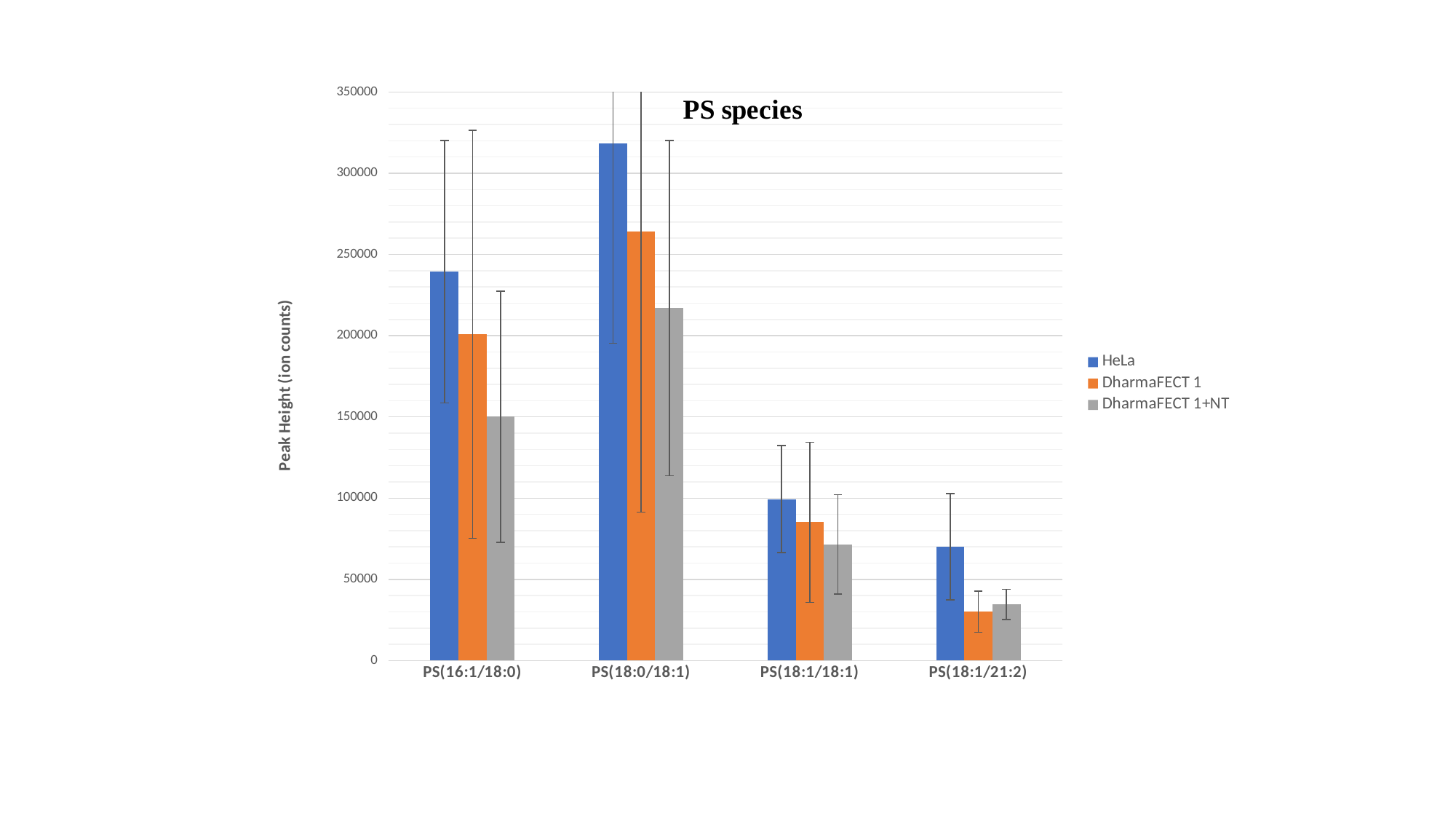

### Chart
| Category | HeLa | DharmaFECT 1 | DharmaFECT 1+NT |
|---|---|---|---|
| PS(16:1/18:0) | 239381.0434163412 | 200847.0831095378 | 150072.6420694988 |
| PS(18:0/18:1) | 318278.7993164063 | 264001.1848958333 | 216937.0551757813 |
| PS(18:1/18:1) | 99383.79256184906 | 85127.6524047851 | 71577.49401855486 |
| PS(18:1/21:2) | 70074.32488769003 | 30172.02751838862 | 34666.10308130483 |

## Slide 10
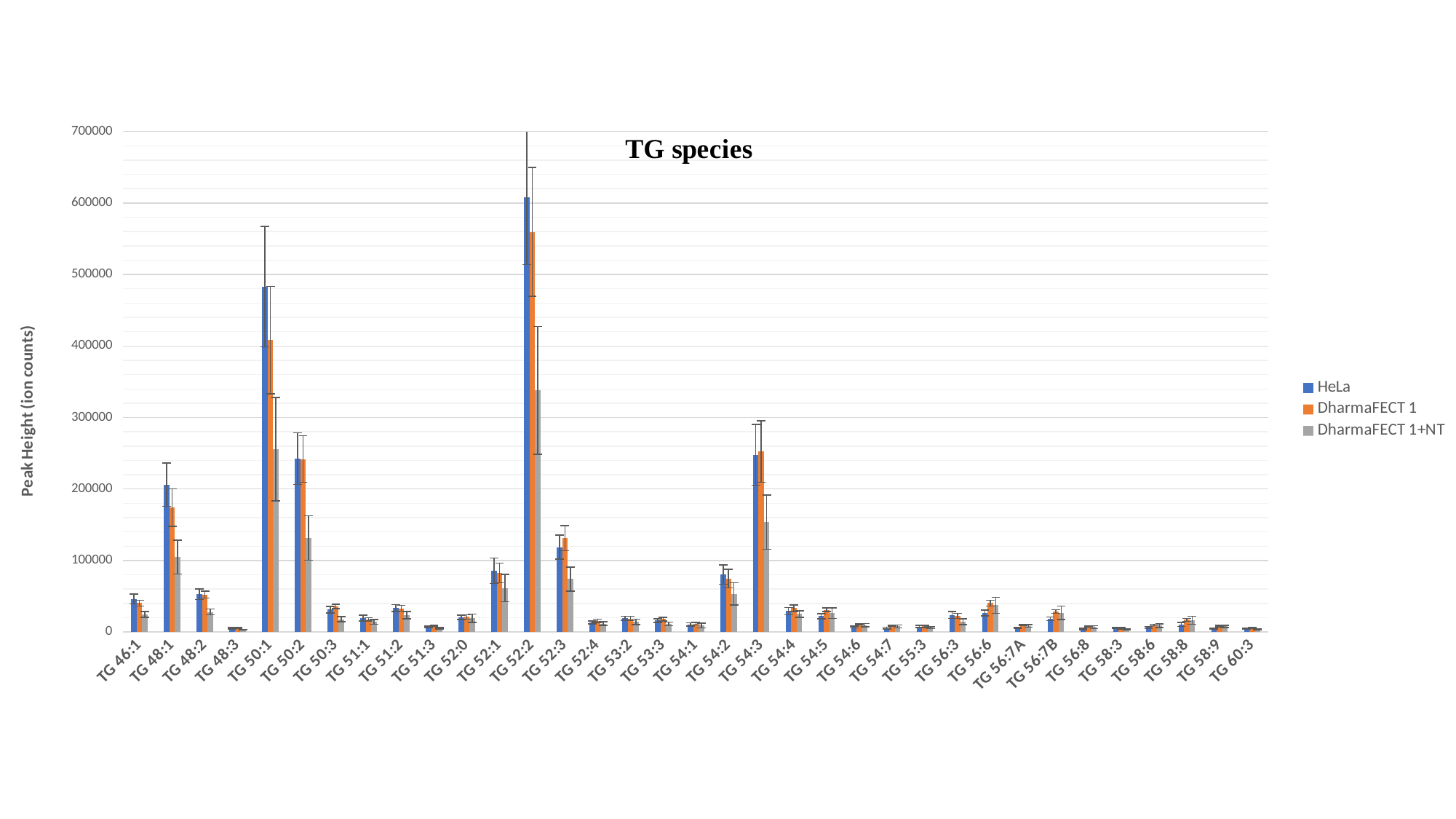

### Chart
| Category | HeLa | DharmaFECT 1 | DharmaFECT 1+NT |
|---|---|---|---|
| TG 46:1 | 46031.5074534153 | 40375.59078362627 | 24764.14257020378 |
| TG 48:1 | 206190.9636213417 | 174182.3138946951 | 104540.2708608792 |
| TG 48:2 | 52932.2409599771 | 52434.72155448053 | 28053.9089891757 |
| TG 48:3 | 5054.428141276042 | 5990.633227030437 | 3333.853736877445 |
| TG 50:1 | 482934.8496581723 | 408301.3506937479 | 255867.2756341428 |
| TG 50:2 | 242444.4469553147 | 241875.0390252945 | 131568.661961929 |
| TG 50:3 | 31259.17651925962 | 35623.29543045619 | 17887.0111837804 |
| TG 51:1 | 19643.78868873357 | 17791.91412850156 | 14303.91593740043 |
| TG 51:2 | 33539.59068175126 | 33178.21885866827 | 23542.2118587087 |
| TG 51:3 | 7067.969656548537 | 8530.989693151405 | 5438.590844835896 |
| TG 52:0 | 20406.2867838542 | 21158.12093098957 | 19318.76420084638 |
| TG 52:1 | 85780.37308650842 | 82657.73093330614 | 61343.92317064137 |
| TG 52:2 | 608073.0804132958 | 559639.46521661 | 337971.2628432447 |
| TG 52:3 | 118695.36856806 | 131312.9315085972 | 74061.72469521823 |
| TG 52:4 | 13451.91544596355 | 15716.6338297526 | 11825.82696533203 |
| TG 53:2 | 19080.29198772127 | 18803.94471737675 | 14262.47717466138 |
| TG 53:3 | 15991.84793141298 | 17995.80188456365 | 11336.29929609742 |
| TG 54:1 | 10755.00074724301 | 11495.4070755826 | 9137.974595959064 |
| TG 54:2 | 80294.11138448896 | 74971.01391376634 | 53557.61999606343 |
| TG 54:3 | 247650.1513904973 | 252259.4298190645 | 153707.9254754524 |
| TG 54:4 | 29197.66053919268 | 33268.56495471366 | 25132.74767109133 |
| TG 54:5 | 22198.11466471357 | 31148.53535970053 | 26337.50764973962 |
| TG 54:6 | 7045.969970703128 | 10533.97680664065 | 9442.004028320316 |
| TG 54:7 | 5467.138020833336 | 9172.76076253256 | 7883.51808675131 |
| TG 55:3 | 7592.715194702148 | 7932.633387247716 | 5978.587435404458 |
| TG 56:3 | 23891.46829485742 | 22420.88054427218 | 14382.57818398372 |
| TG 56:6 | 26647.82881673178 | 40504.48999023438 | 37355.27823893232 |
| TG 56:7A | 5853.059010823567 | 10210.24654134116 | 8672.827392578129 |
| TG 56:7B | 18431.58834838866 | 28790.71990966798 | 26867.02997843425 |
| TG 56:8 | 4276.756245930988 | 7453.460327148438 | 6779.819295247398 |
| TG 58:3 | 5506.920687357585 | 5310.79337819417 | 3428.55540974935 |
| TG 58:6 | 6101.497528076172 | 9732.14717610677 | 8799.732930501295 |
| TG 58:8 | 10551.73927815756 | 17124.31042480468 | 16352.31949869793 |
| TG 58:9 | 4382.31229909261 | 8187.059377034503 | 7880.19269816081 |
| TG 60:3 | 5031.121461290445 | 5420.141426141453 | 3659.458913741193 |

## Slide 11
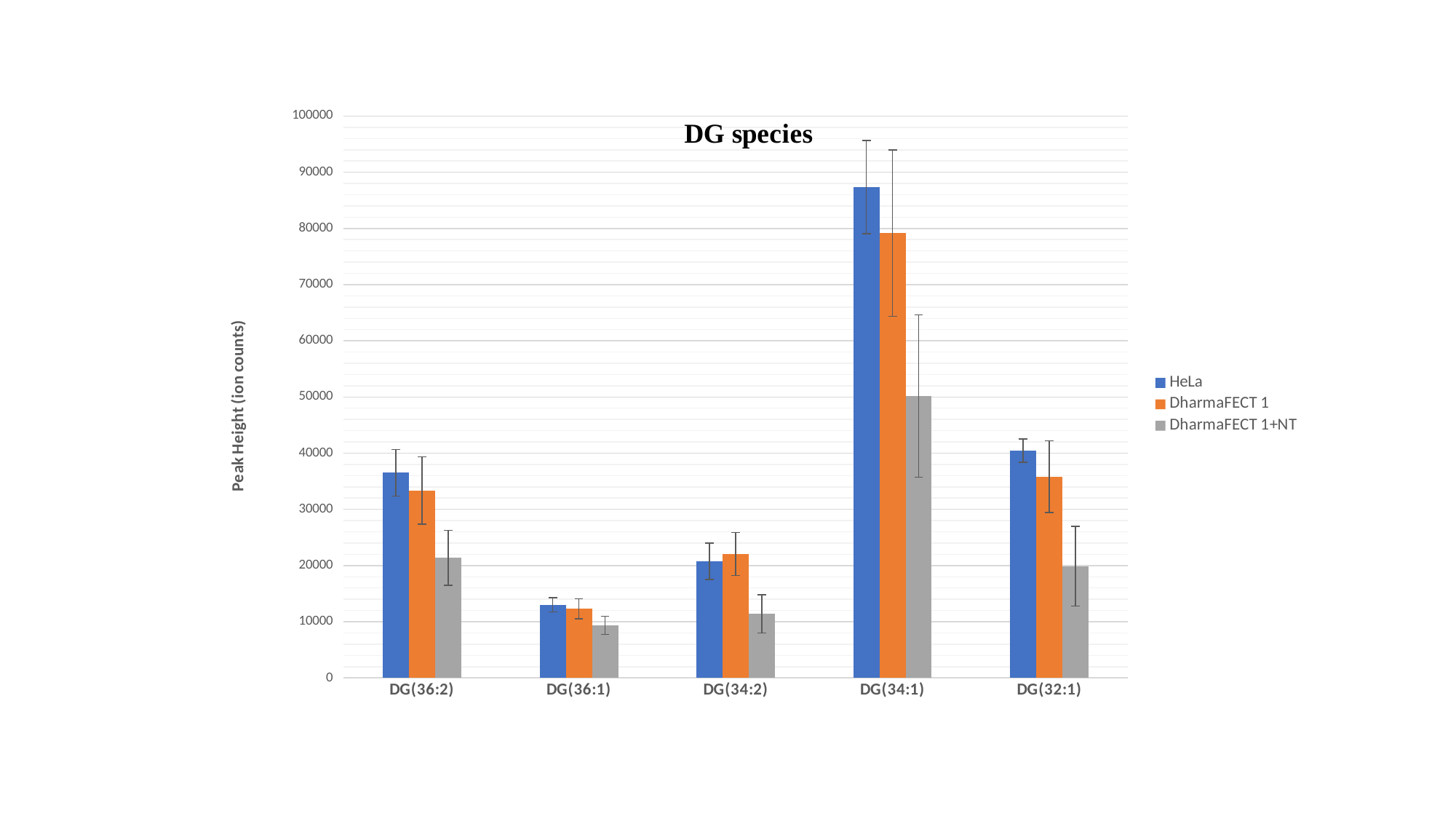

### Chart
| Category | HeLa | DharmaFECT 1 | DharmaFECT 1+NT |
|---|---|---|---|
| DG(36:2) | 36501.02788289385 | 33349.82594807941 | 21378.20845540367 |
| DG(36:1) | 13033.2933451335 | 12301.57727813722 | 9346.104690551747 |
| DG(34:2) | 20761.17407226564 | 22041.7265930176 | 11396.49326578777 |
| DG(34:1) | 87366.25263468416 | 79166.70639038087 | 50177.46882120769 |
| DG(32:1) | 40453.12814839681 | 35819.91347757976 | 19882.90916697183 |
