# Supplementary material for: RNAi Transfection Results in Lipidome Changes
Source: Proteomics. 2019 Jun 13;19(13):1800298. doi: 10.1002/pmic.201800298 (PMC6617754; doi:10.1002/pmic.201800298)
Supplement: Supplementary file 6 — Supporting Information [file PMIC-19-na-s006.pptx]

## Slide 1
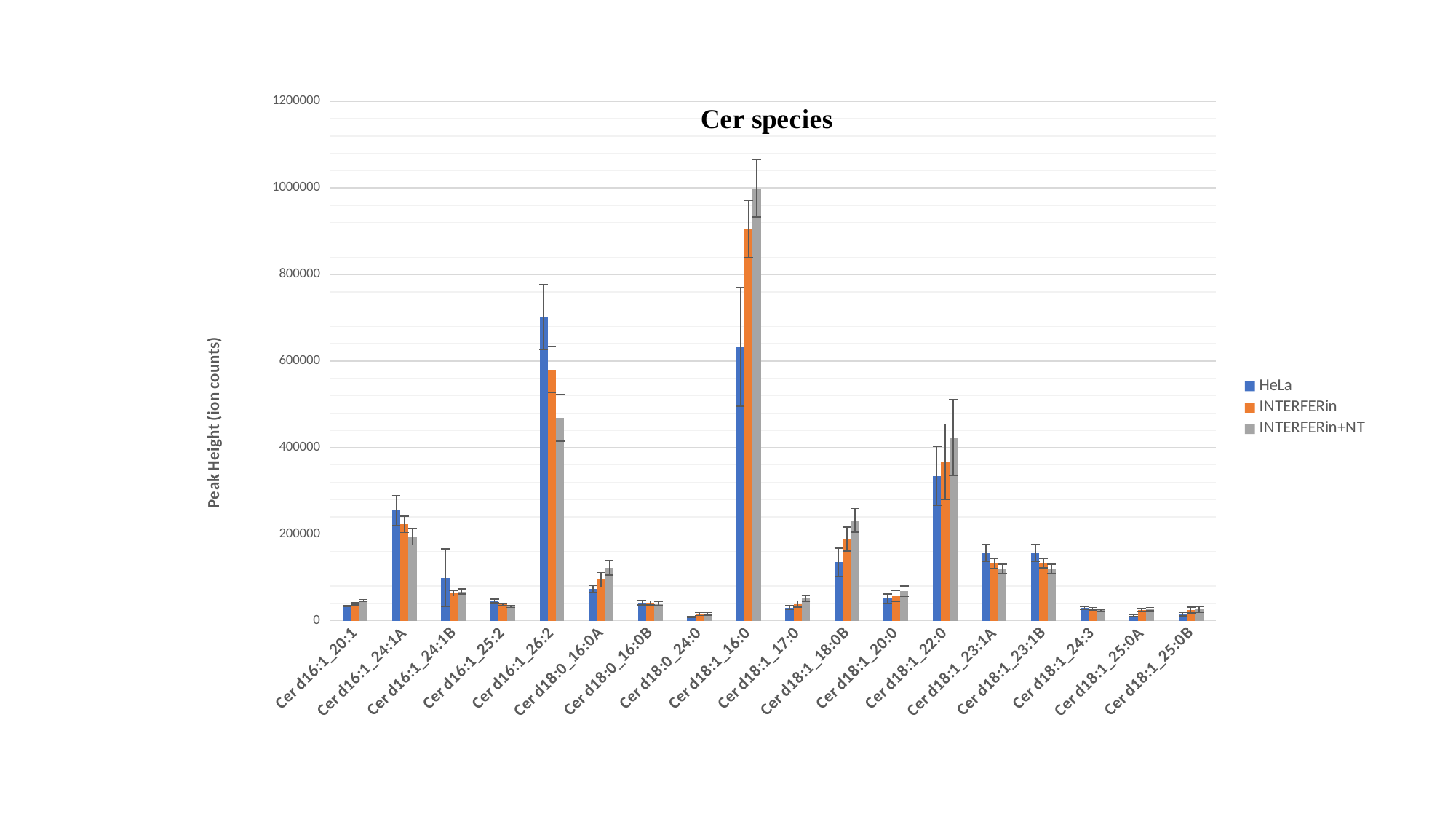

### Chart
| Category | HeLa | INTERFERin | INTERFERin+NT |
|---|---|---|---|
| Cer d16:1_20:1 | 34034.8010457357 | 38878.61853027345 | 46422.67427571616 |
| Cer d16:1_24:1A | 254574.9905772725 | 222531.8655531433 | 193943.8024250892 |
| Cer d16:1_24:1B | 98714.388988388 | 63716.22080336483 | 67425.31109068618 |
| Cer d16:1_25:2 | 45545.23365676342 | 37880.22392950347 | 32917.96456360682 |
| Cer d16:1_26:2 | 701937.920136144 | 580406.1407100968 | 468726.1490705329 |
| Cer d18:0_16:0A | 72588.21943410234 | 94436.5791651407 | 122318.8656336466 |
| Cer d18:0_16:0B | 41802.29223632815 | 40941.02490234377 | 39493.42346191408 |
| Cer d18:0_24:0 | 8501.407020568846 | 15558.70658874512 | 15918.76727803548 |
| Cer d18:1_16:0 | 633076.467122396 | 904883.1997070316 | 999377.6855468749 |
| Cer d18:1_17:0 | 29748.6605430589 | 38187.52296067987 | 51427.41894458822 |
| Cer d18:1_18:0B | 134932.927368164 | 188489.4532877603 | 231791.6950276693 |
| Cer d18:1_20:0 | 51321.72212055592 | 57006.05618528341 | 68151.31035579192 |
| Cer d18:1_22:0 | 334283.7326463625 | 367108.494597751 | 423628.858188359 |
| Cer d18:1_23:1A | 156691.8027727515 | 131714.1382026077 | 119451.661035937 |
| Cer d18:1_23:1B | 156963.1562044277 | 133411.311218324 | 119540.7766658254 |
| Cer d18:1_24:3 | 29090.32889811197 | 27509.10493977865 | 23973.84815470377 |
| Cer d18:1_25:0A | 11592.24300130209 | 24969.09871419274 | 26852.4656575521 |
| Cer d18:1_25:0B | 15053.4719543457 | 24358.7029622396 | 25469.4794921875 |

## Slide 2
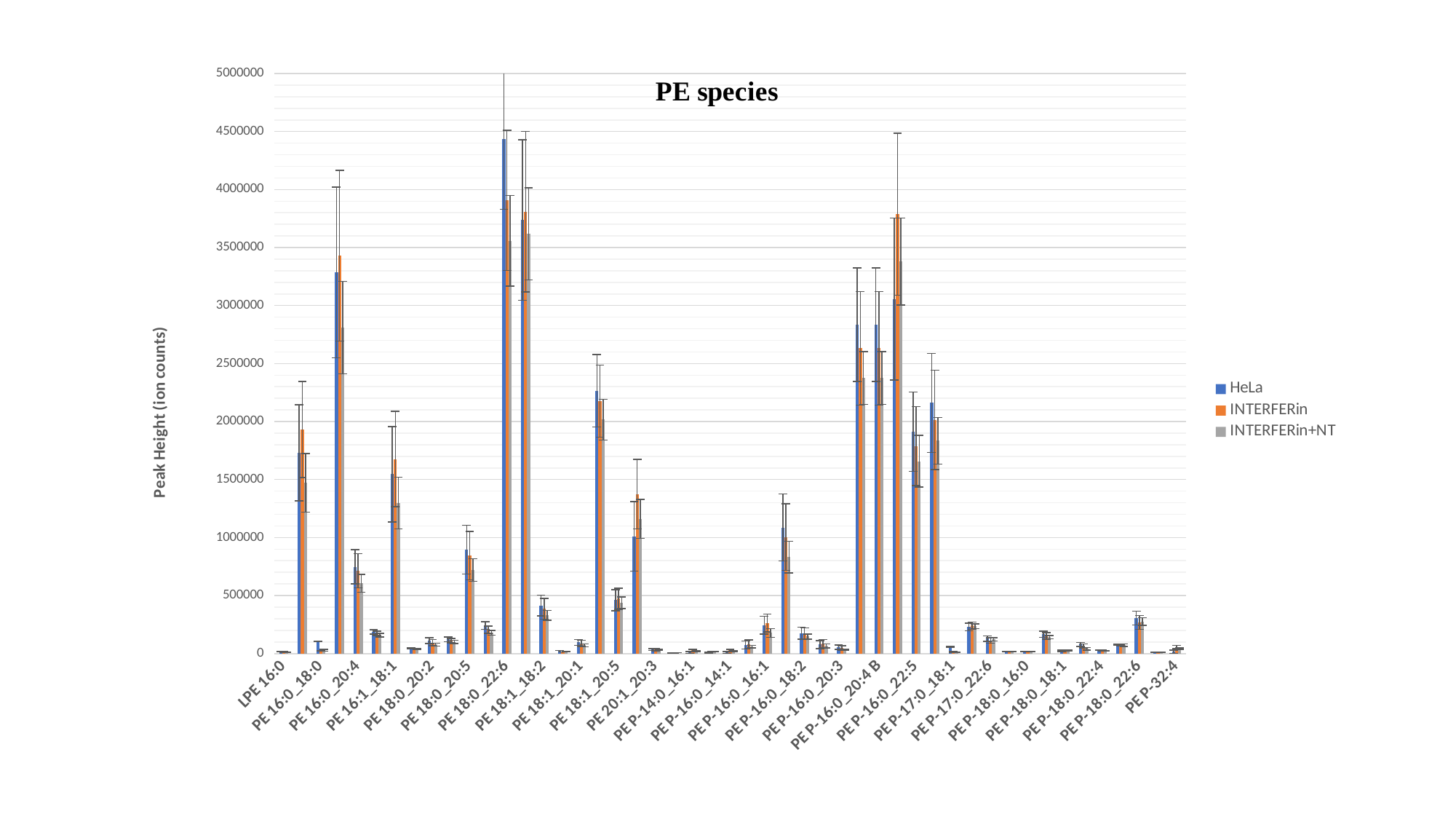

### Chart
| Category | HeLa | INTERFERin | INTERFERin+NT |
|---|---|---|---|
| LPE 16:0 | 16338.15492757162 | 14068.1612065633 | 11052.74262746176 |
| PE 16:0_16:1 | 1730643.52205404 | 1931415.18001302 | 1470636.35001628 |
| PE 16:0_18:0 | 104617.9330647787 | 23792.40431722007 | 27392.56689453125 |
| PE 16:0_18:1 | 3285470.81835937 | 3429727.73567708 | 2809879.17708333 |
| PE 16:0_20:4 | 747781.2091471354 | 714678.5039062503 | 604745.463541667 |
| PE 16:0_20:5 | 187387.072591146 | 173086.9786783855 | 159053.6891276041 |
| PE 16:1_18:1 | 1545120.3282453 | 1676737.2178197 | 1297136.09755769 |
| PE 17:0_20:4 | 46534.60660807292 | 41593.95458984375 | 40773.3642578125 |
| PE 18:0_20:2 | 111104.6025390625 | 93707.6426595052 | 76857.13362630218 |
| PE 18:0_20:3 | 122229.9427083334 | 110779.4106445313 | 100111.8470052084 |
| PE 18:0_20:5 | 895808.6521949846 | 844261.0866156599 | 719372.1965917883 |
| PE 18:0_22:5 | 242538.6790184791 | 204891.4089805715 | 177532.7178180208 |
| PE 18:0_22:6 | 4434343.6532387 | 3906668.98412641 | 3557723.0057615 |
| PE 18:1_18:1 | 3736557.69821481 | 3809638.60652009 | 3618169.40412903 |
| PE 18:1_18:2 | 413945.7594904778 | 386341.2314183772 | 329540.4426721973 |
| PE 18:1_20:0 | 26304.77189127605 | 13783.20703125001 | 15900.45312500001 |
| PE 18:1_20:1 | 96121.81510416682 | 88045.19807942715 | 70793.26725260417 |
| PE 18:1_20:3 | 2265893.14863182 | 2175700.98741287 | 2016097.88142764 |
| PE 18:1_20:5 | 459923.0401882352 | 472183.6108620625 | 436233.0920144425 |
| PE 18:1_22:6 | 1009878.16310169 | 1373686.14370234 | 1160785.32440825 |
| PE 20:1_20:3 | 35145.13069661459 | 32991.5174153646 | 31711.3818359375 |
| PE 20:5_22:6 | 6012.51355489095 | 6196.46160888672 | 8034.728464762372 |
| PE P-14:0_16:1 | 10436.10793558757 | 28849.77770996096 | 21089.63774617513 |
| PE P-16:0_12:0 | 7094.118489583336 | 16288.59927368163 | 17261.04320271812 |
| PE P-16:0_14:1 | 9208.379659016924 | 27426.70271809895 | 20282.24287923178 |
| PE P-16:0_16:0 | 73048.35461425783 | 81718.74558512375 | 58150.68328857421 |
| PE P-16:0_16:1 | 244696.6805419925 | 262265.6938476565 | 177939.6459147137 |
| PE P-16:0_18:1 | 1086415.39163606 | 1004234.69669311 | 831408.846724122 |
| PE P-16:0_18:2 | 175734.8177083333 | 169983.00390625 | 146551.828125 |
| PE P-16:0_20:3 | 77064.37369791667 | 85802.29752604167 | 66303.15690104167 |
| PE P-16:0_20:3 | 54849.63460286459 | 48578.2451985677 | 34538.90478515627 |
| PE P-16:0_20:4 A | 2834158.65234375 | 2631854.859375 | 2375085.01171875 |
| PE P-16:0_20:4 B | 2834158.65234375 | 2631854.859375 | 2375200.27929688 |
| PE P-16:0_20:5 | 3057230.30338542 | 3786507.57682292 | 3380396.52473958 |
| PE P-16:0_22:5 | 1912440.12044271 | 1788366.29752604 | 1657846.15983073 |
| PE P-16:0_22:6 | 2160933.45706138 | 2014661.97914176 | 1834847.23142123 |
| PE P-17:0_18:1 | 56153.07779947917 | 11944.5283203125 | 10831.17643229167 |
| PE P-17:0_20:4 | 228867.8110351565 | 240714.0838216148 | 235214.9408365889 |
| PE P-17:0_22:6 | 127448.6168619792 | 112479.9967447917 | 122732.2469075523 |
| PE P-18:0_16:0 | 16456.90474446618 | 17783.50216674806 | 18874.85072835286 |
| PE P-18:0_16:0 | 16367.46724446618 | 17444.71444702152 | 18731.8462931315 |
| PE P-18:0_18:1 | 166117.5030110678 | 150882.2923990885 | 141724.8661295573 |
| PE P-18:0_18:1 | 22896.76778157553 | 27498.49226888021 | 24980.68652343751 |
| PE P-18:0_20:3 | 78460.49397786456 | 66431.40185546874 | 40253.6845703125 |
| PE P-18:0_22:4 | 29726.39306640626 | 26539.70638020838 | 21033.21728515627 |
| PE P-18:0_22:5 | 76724.7744140625 | 73184.82877604169 | 71846.03190104167 |
| PE P-18:0_22:6 | 305179.2446670614 | 269543.0481242763 | 275643.1150390869 |
| PE P-20:0_22:6 | 12486.98472595215 | 12378.52436574299 | 12880.68158721925 |
| PE P-32:4 | 15208.21004231772 | 55068.65193684898 | 42914.54736328125 |

## Slide 3
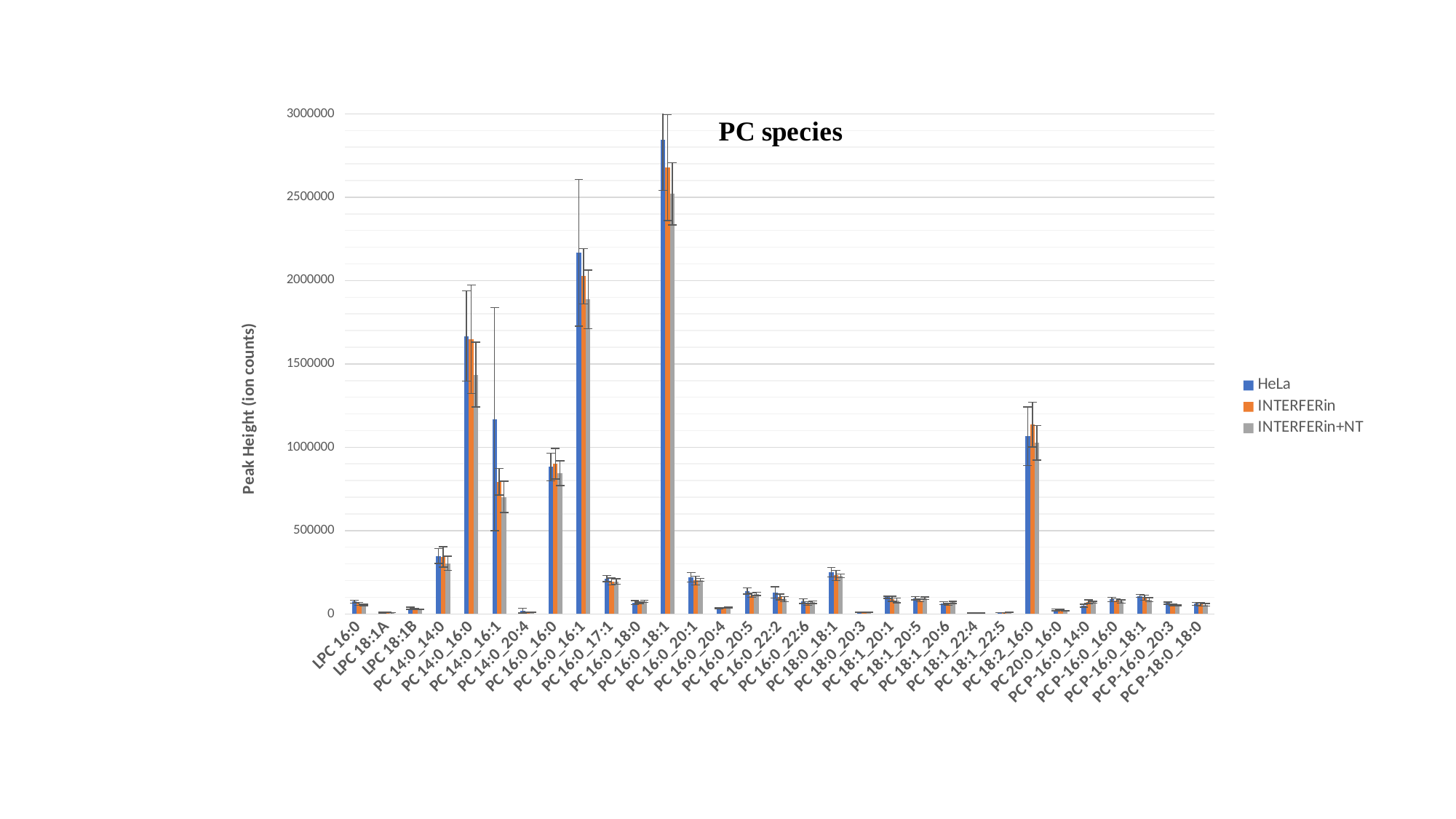

### Chart
| Category | HeLa | INTERFERin | INTERFERin+NT |
|---|---|---|---|
| LPC 16:0 | 73942.47220865889 | 60722.80289713543 | 54349.06795247401 |
| LPC 18:1A | 9268.26896158855 | 8060.152669270847 | 7310.140401204427 |
| LPC 18:1B | 33763.42951456704 | 29061.85926310221 | 26965.54232788086 |
| PC 14:0_14:0 | 347983.8337166666 | 342175.41605 | 303131.9974000001 |
| PC 14:0_16:0 | 1667332.09783333 | 1647862.14816667 | 1435945.16366667 |
| PC 14:0_16:1 | 1169168.4916 | 792746.2591333333 | 702209.9767333334 |
| PC 14:0_20:4 | 19841.67504749999 | 7930.429850333333 | 9225.861653166663 |
| PC 16:0_16:0 | 881403.5143166664 | 900228.3001833335 | 843670.41015 |
| PC 16:0_16:1 | 2166451.2475 | 2025964.2395 | 1887483.69283333 |
| PC 16:0_17:1 | 212230.4459833333 | 194911.7011666667 | 194494.6888 |
| PC 16:0_18:0 | 67789.12141833336 | 66797.44140666665 | 75560.314455 |
| PC 16:0_18:1 | 2844580.8145 | 2677776.2475 | 2520869.232 |
| PC 16:0_20:1 | 219351.8219333333 | 200541.9736333333 | 203669.4502 |
| PC 16:0_20:4 | 34814.90364666666 | 35990.52238 | 38495.75960166666 |
| PC 16:0_20:5 | 137767.9210666666 | 111821.9081983333 | 120700.4576833333 |
| PC 16:0_22:2 | 129061.06846 | 102010.1958766667 | 89469.27775833337 |
| PC 16:0_22:6 | 76086.48990833334 | 62894.238605 | 69285.48209833333 |
| PC 18:0_18:1 | 250239.2712 | 230628.0802333333 | 227945.7924666666 |
| PC 18:0_20:3 | 9509.496133833329 | 8857.895914166664 | 9837.595662666665 |
| PC 18:1_20:1 | 99986.65998166664 | 90741.79638166663 | 81416.91894999999 |
| PC 18:1_20:5 | 93276.40055833333 | 81481.34554000001 | 94396.90982666664 |
| PC 18:1_20:6 | 64133.40104333332 | 56730.924805 | 68965.277345 |
| PC 18:1_22:4 | 5121.754679500001 | 5144.582479 | 6876.500529 |
| PC 18:1_22:5 | 7792.215820333334 | 7363.147745666666 | 8797.5525715 |
| PC 18:2_16:0 | 1065564.45576667 | 1135965.09633333 | 1026043.69408333 |
| PC 20:0_16:0 | 24318.10196833332 | 24211.98335833333 | 18046.32942833333 |
| PC P-16:0_14:0 | 50041.197835 | 72949.61108333334 | 70834.26660166663 |
| PC P-16:0_16:0 | 86962.51041166665 | 81760.43066333333 | 75060.00423166664 |
| PC P-16:0_18:1 | 107997.1454466667 | 97361.07597499999 | 85523.81452833334 |
| PC P-16:0_20:3 | 64553.77189166665 | 54726.43668666665 | 51854.94181333334 |
| PC P-18:0_18:0 | 60280.19385 | 57132.53727333333 | 54549.77832 |

## Slide 4
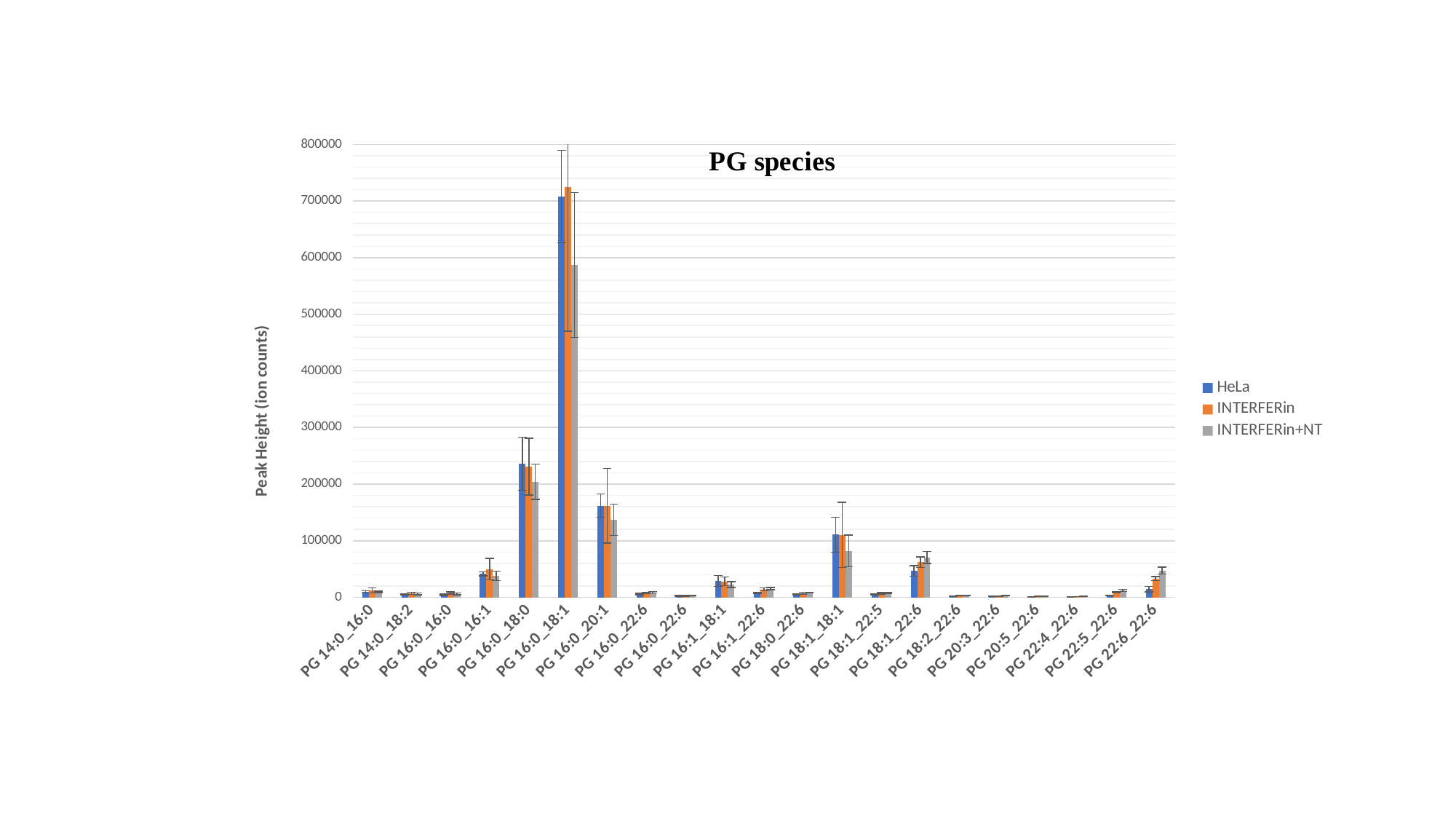

### Chart
| Category | HeLa | INTERFERin | INTERFERin+NT |
|---|---|---|---|
| PG 14:0_16:0 | 9824.205764770508 | 12181.43323771159 | 9601.958343505865 |
| PG 14:0_18:2 | 5157.03210449219 | 7061.111546834312 | 6114.05487060547 |
| PG 16:0_16:0 | 4569.669433593752 | 7881.797017415372 | 6109.338724772136 |
| PG 16:0_16:1 | 41546.935546875 | 49699.17781575523 | 38049.99259440106 |
| PG 16:0_18:0 | 236036.1309254247 | 230713.107656959 | 204120.3703951648 |
| PG 16:0_18:1 | 707924.9044290186 | 724938.0233573132 | 587160.5209913574 |
| PG 16:0_20:1 | 161876.4934082034 | 161863.1064453127 | 137150.9711100263 |
| PG 16:0_22:6 | 5465.35971069336 | 7444.137919108074 | 8825.831370035809 |
| PG 16:0_22:6 | 2814.91343688965 | 2881.360590616862 | 3327.38375854492 |
| PG 16:1_18:1 | 28935.1745223999 | 28172.0129699707 | 22245.51516723633 |
| PG 16:1_22:6 | 8161.079399108889 | 14071.96001688638 | 15222.77424621582 |
| PG 18:0_22:6 | 5200.576182047526 | 7216.232686360677 | 8304.752583821617 |
| PG 18:1_18:1 | 110589.8548177085 | 110379.7035319011 | 81868.61234537762 |
| PG 18:1_22:5 | 4992.963022867841 | 6841.503153483071 | 7914.36334228516 |
| PG 18:1_22:6 | 46441.87338256836 | 61857.33666483562 | 70426.07859039305 |
| PG 18:2_22:6 | 2151.46863301595 | 3121.300404866537 | 3333.555984497072 |
| PG 20:3_22:6 | 1964.297083536783 | 2348.818237304689 | 2736.080647786457 |
| PG 20:5_22:6 | 954.7708638509121 | 1702.453852335613 | 2162.792747497558 |
| PG 22:4_22:6 | 949.9395345052093 | 1306.179026285808 | 1737.728515625002 |
| PG 22:5_22:6 | 3359.506490071615 | 8565.356160481773 | 12309.62280273439 |
| PG 22:6_22:6 | 14204.34393310545 | 32797.26570638022 | 47195.858194987 |

## Slide 5
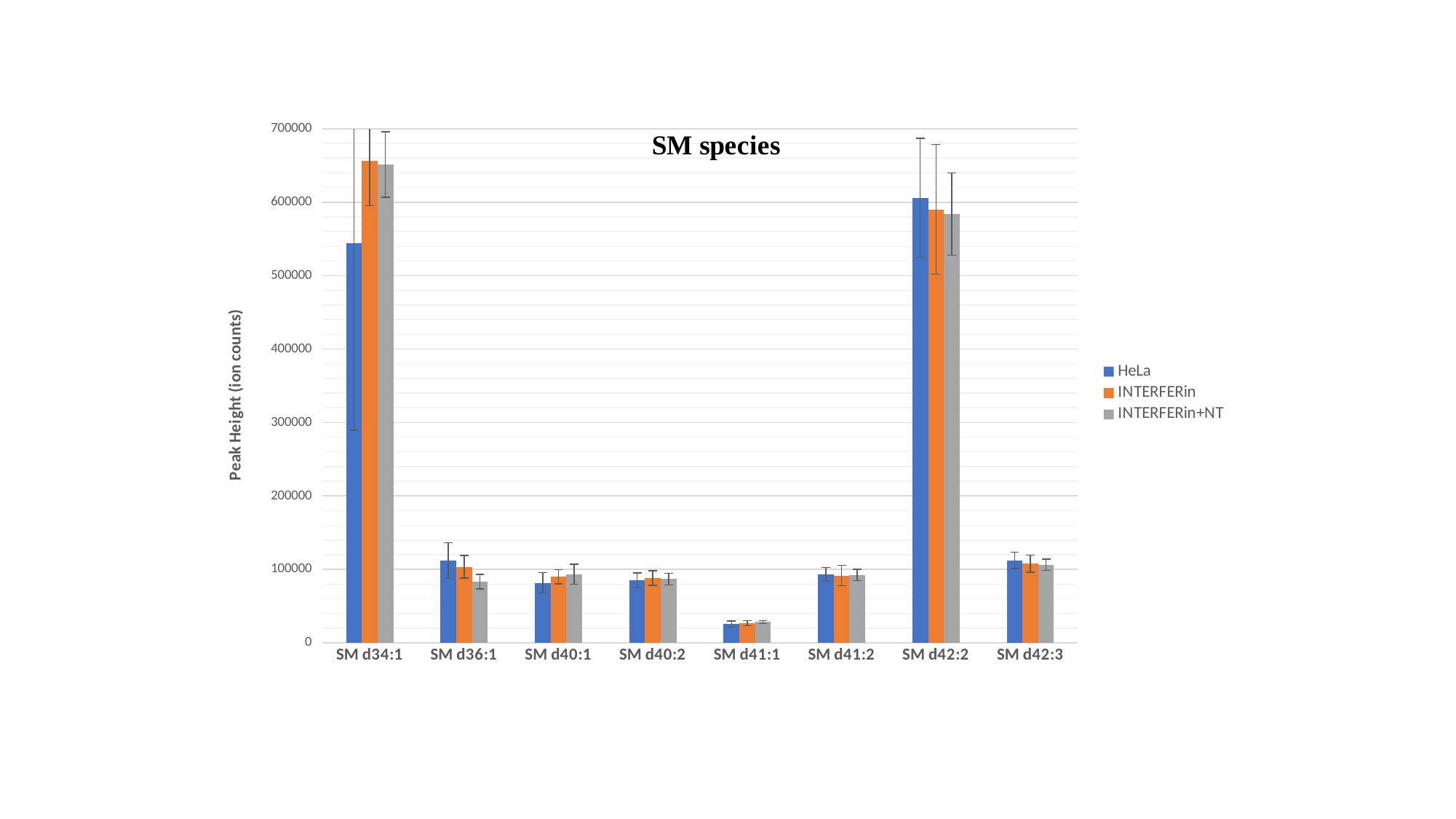

### Chart
| Category | HeLa | INTERFERin | INTERFERin+NT |
|---|---|---|---|
| SM d34:1 | 543916.1362266405 | 656013.8643165288 | 651215.7800084186 |
| SM d36:1 | 112018.8136698406 | 103558.4870198568 | 83537.380607605 |
| SM d40:1 | 81706.73860677083 | 89845.47037760417 | 93534.23535156249 |
| SM d40:2 | 85018.77055314946 | 88400.68120054605 | 86811.06098182146 |
| SM d41:1 | 25386.62174479167 | 26801.30192057291 | 28274.19482421875 |
| SM d41:2 | 93027.39897212916 | 91616.61686129925 | 92430.07848887805 |
| SM d42:2 | 605987.6627139584 | 590267.5319369622 | 583874.0866371375 |
| SM d42:3 | 112411.5790201824 | 107837.8751627607 | 106354.2021077474 |

## Slide 6
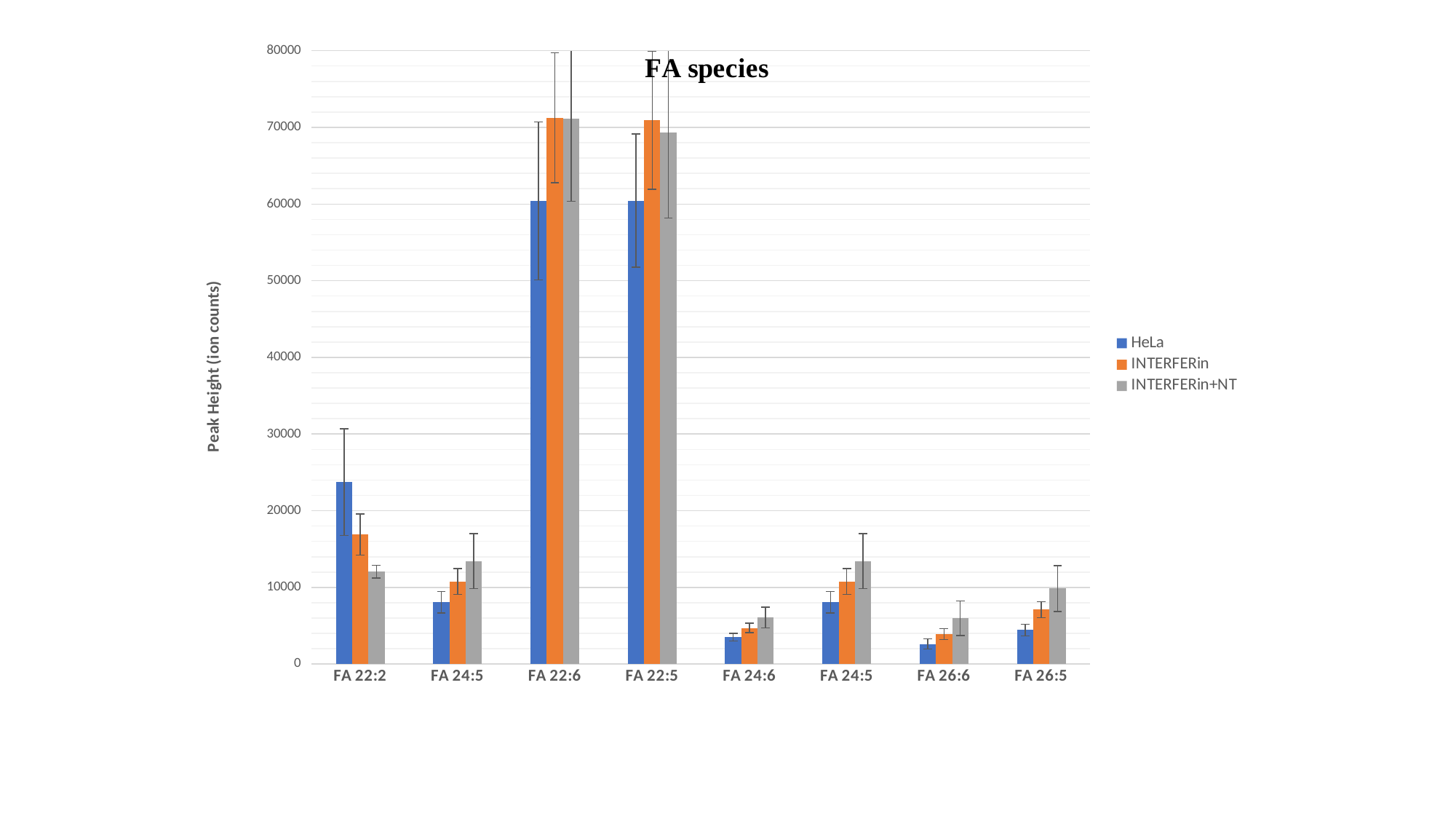

### Chart
| Category | HeLa | INTERFERin | INTERFERin+NT |
|---|---|---|---|
| FA 22:2 | 23720.05323678684 | 16901.99654234436 | 12036.7452295989 |
| FA 24:5 | 8062.206817525625 | 10775.90400390625 | 13447.9548828125 |
| FA 22:6 | 60408.26750370203 | 71241.92437494354 | 71136.5690161762 |
| FA 22:5 | 60452.26723056265 | 70915.24655852189 | 69378.6376159137 |
| FA 24:6 | 3503.832773000388 | 4718.78950750341 | 6069.220905653194 |
| FA 24:5 | 8063.824577681813 | 10775.90400390625 | 13447.9548828125 |
| FA 26:6 | 2620.657972373664 | 3901.116618771718 | 5956.408263986212 |
| FA 26:5 | 4442.992187199174 | 7097.085270600661 | 9864.685527963491 |

## Slide 7
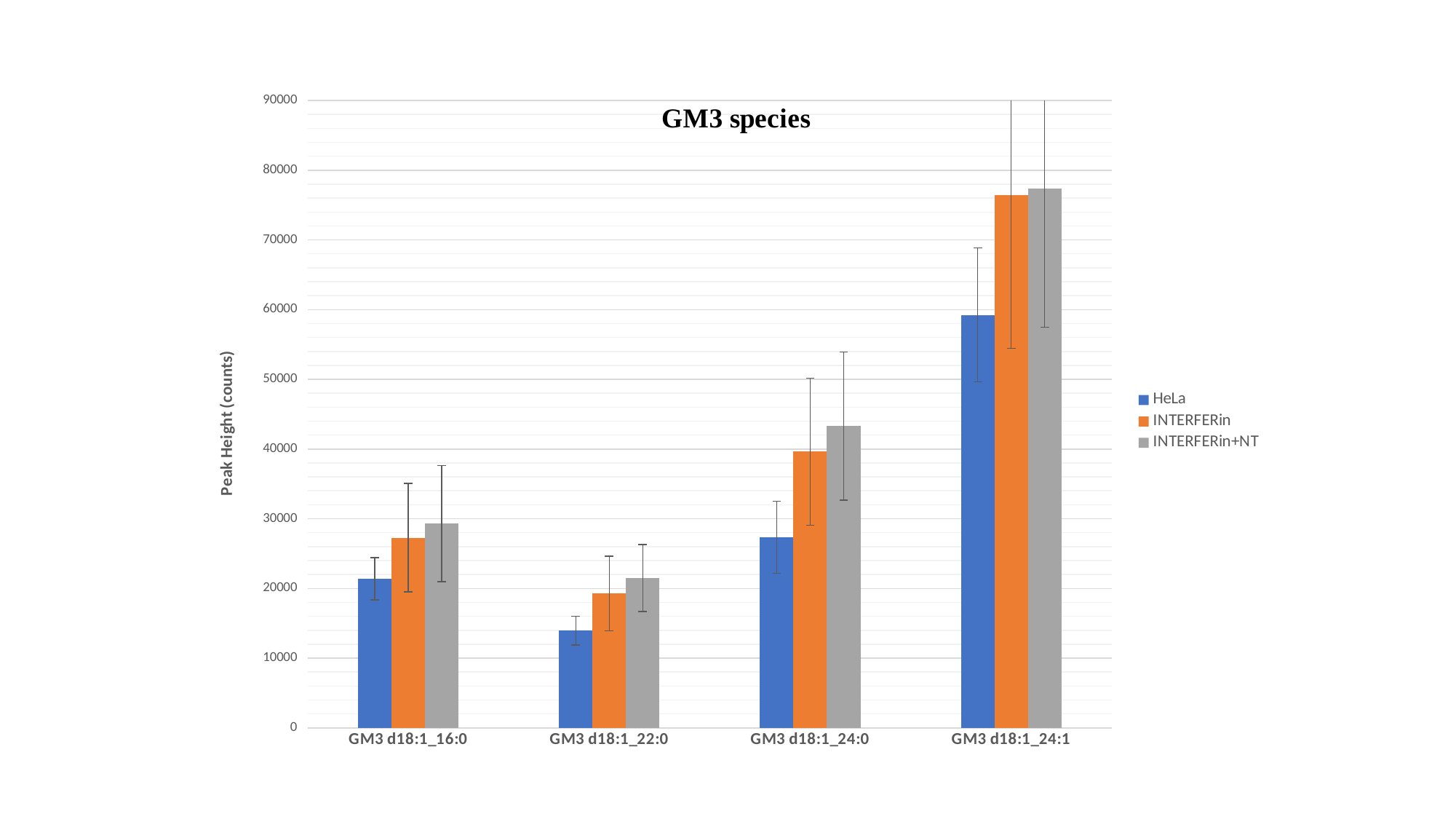

### Chart
| Category | HeLa | INTERFERin | INTERFERin+NT |
|---|---|---|---|
| GM3 d18:1_16:0 | 21399.83772428033 | 27280.94009814554 | 29285.12269452552 |
| GM3 d18:1_22:0 | 13939.40581211863 | 19266.40659453229 | 21499.57640332522 |
| GM3 d18:1_24:0 | 27339.88179329067 | 39609.98735070358 | 43296.18156969243 |
| GM3 d18:1_24:1 | 59252.68595317265 | 76449.39913426967 | 77327.84896827063 |

## Slide 8
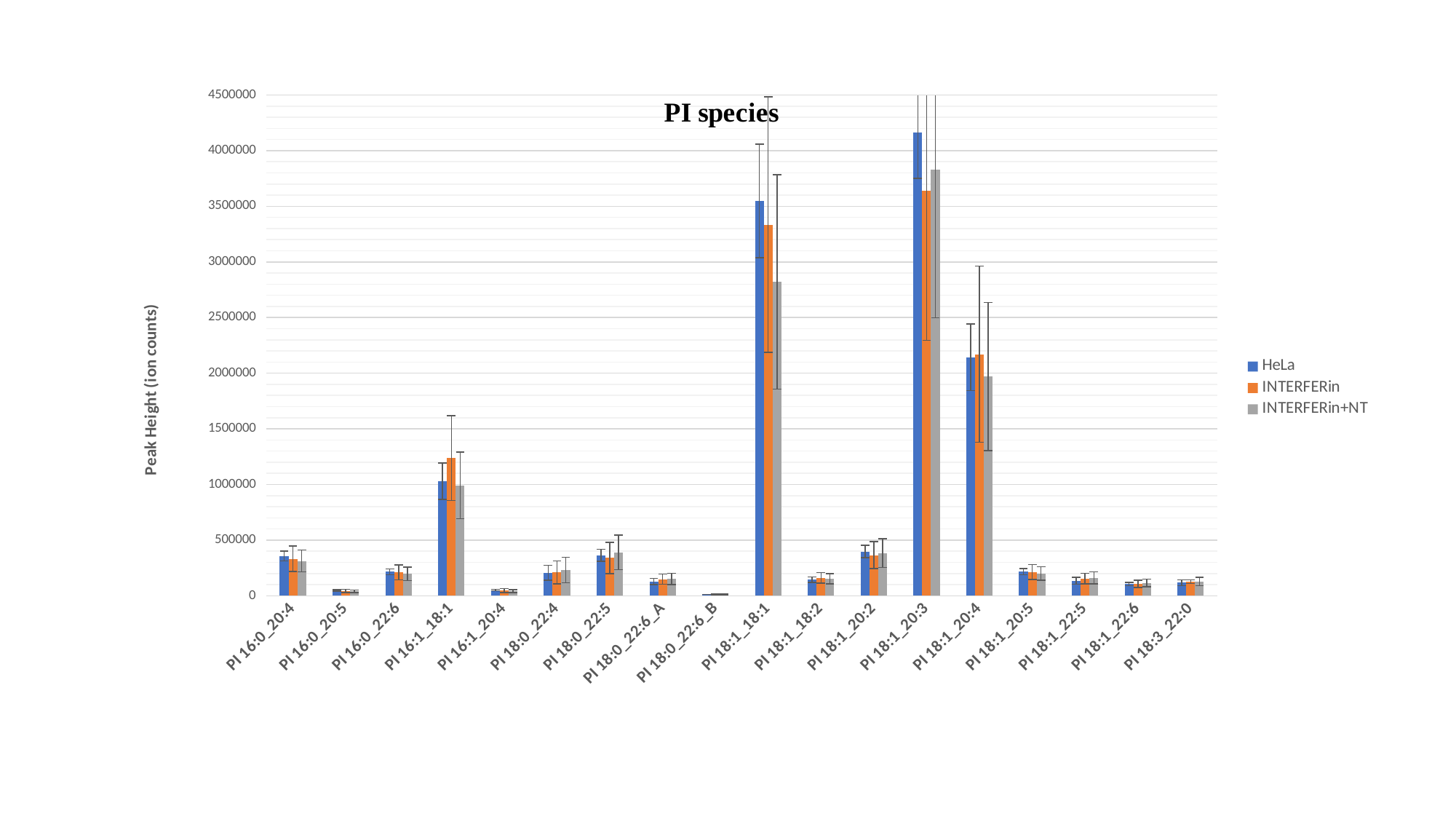

### Chart
| Category | HeLa | INTERFERin | INTERFERin+NT |
|---|---|---|---|
| PI 16:0_20:4 | 358227.597133201 | 332138.993112401 | 312017.1930355428 |
| PI 16:0_20:5 | 48515.38492838544 | 43371.64274088541 | 39899.522257487 |
| PI 16:0_22:6 | 215643.8873697917 | 211371.8698527019 | 197796.8169555666 |
| PI 16:1_18:1 | 1029521.95980813 | 1236623.24867164 | 991591.0911503216 |
| PI 16:1_20:4 | 51564.38558247356 | 46325.75382503812 | 42461.26550553046 |
| PI 18:0_22:4 | 207601.2876610385 | 210380.5794976216 | 231361.1525792782 |
| PI 18:0_22:5 | 364474.2060908724 | 340134.3948230009 | 390134.467313867 |
| PI 18:0_22:6_A | 128241.3655598961 | 148789.8155110678 | 150960.0128580733 |
| PI 18:0_22:6_B | 12645.45764160158 | 11848.76330566407 | 13147.60978190107 |
| PI 18:1_18:1 | 3548132.40644794 | 3334469.80858182 | 2820881.81121669 |
| PI 18:1_18:2 | 143641.2954915365 | 161119.9074300133 | 153020.1744384767 |
| PI 18:1_20:2 | 398184.6356819682 | 365508.4356757992 | 384868.5099101427 |
| PI 18:1_20:3 | 4165767.00097054 | 3636844.36764 | 3830743.05687922 |
| PI 18:1_20:4 | 2143156.44463267 | 2171011.47851106 | 1970233.80186334 |
| PI 18:1_20:5 | 217558.6338663537 | 213059.0115342462 | 199965.0560601633 |
| PI 18:1_22:5 | 135381.0026616453 | 155075.2807227124 | 160189.6796169127 |
| PI 18:1_22:6 | 105252.7181266538 | 107526.8005224448 | 115234.4104311508 |
| PI 18:3_22:0 | 118170.889712981 | 127793.3614571534 | 127922.8443189453 |

## Slide 9
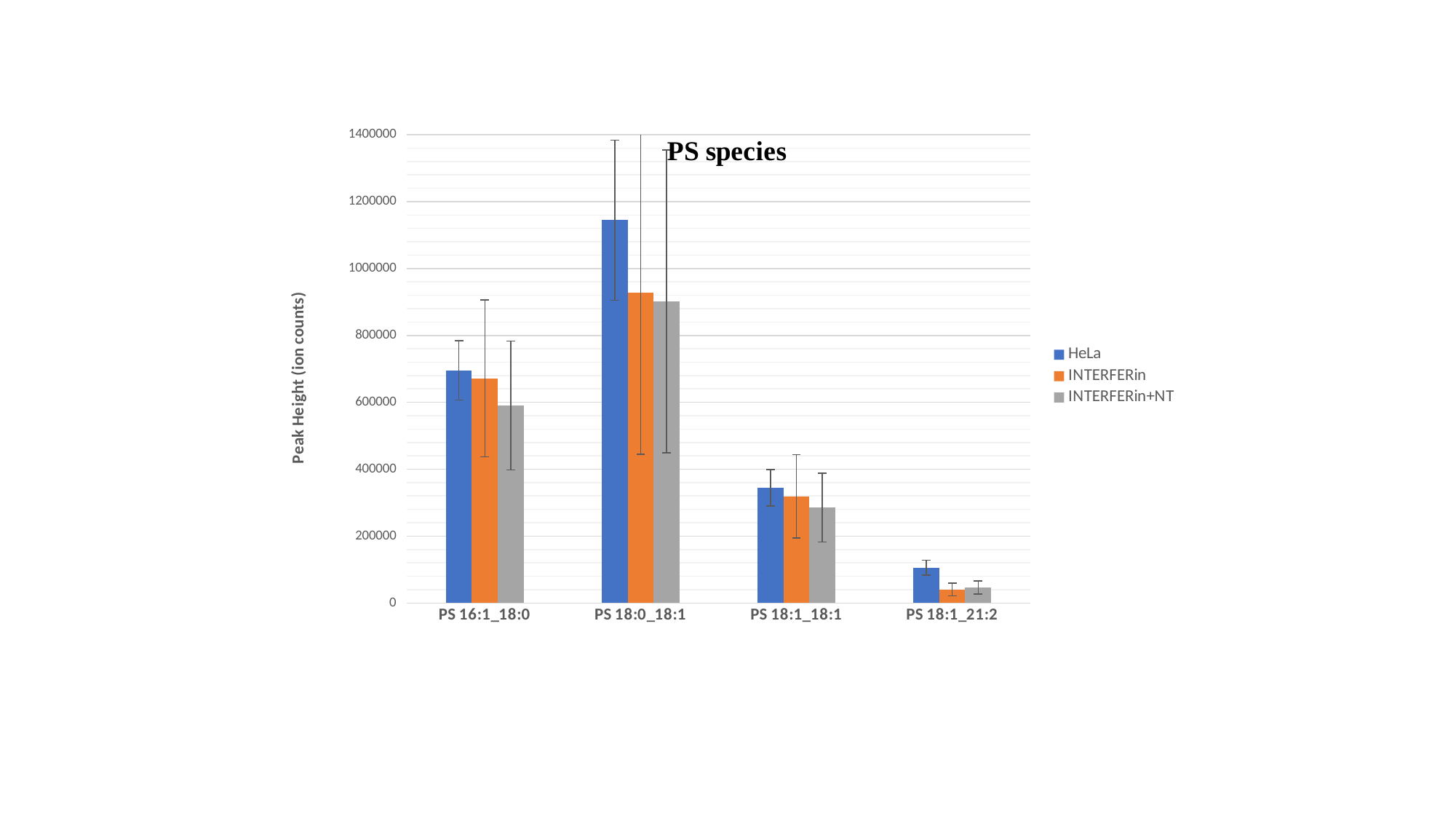

### Chart
| Category | HeLa | INTERFERin | INTERFERin+NT |
|---|---|---|---|
| PS 16:1_18:0 | 695579.930338542 | 671385.4001464844 | 590538.8226318364 |
| PS 18:0_18:1 | 1144463.31901042 | 927900.7451171874 | 901669.2408854167 |
| PS 18:1_18:1 | 344049.5275065105 | 319097.7773437502 | 285062.4685058595 |
| PS 18:1_21:2 | 105396.2087661313 | 40555.03145427519 | 46440.88275376365 |

## Slide 10
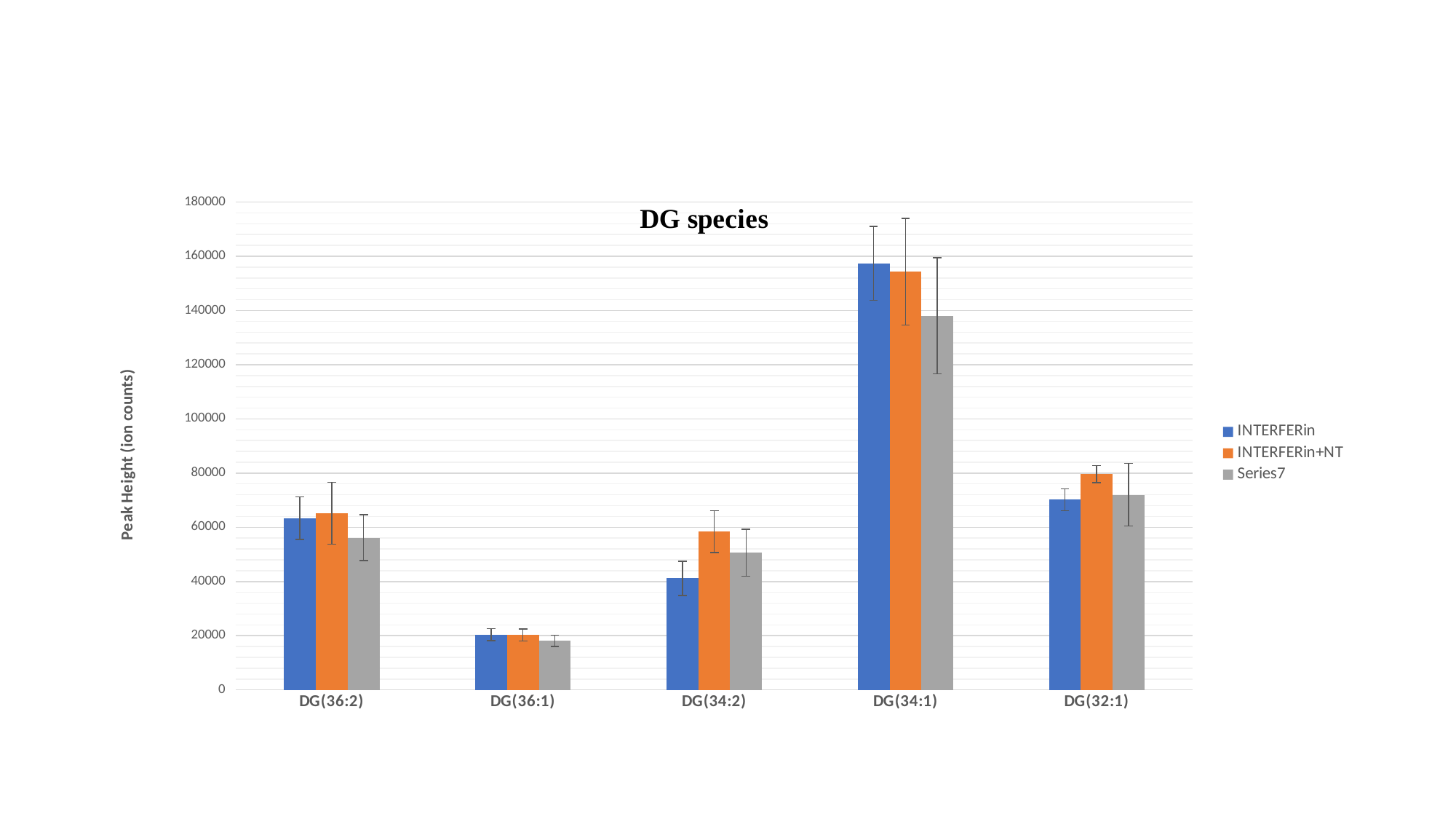

### Chart
| Category | INTERFERin | INTERFERin+NT | |
|---|---|---|---|
| DG(36:2) | 63357.22926839196 | 65210.79441324871 | 56169.03607177737 |
| DG(36:1) | 20388.48427073163 | 20267.47642517092 | 18116.24364217122 |
| DG(34:2) | 41183.01519775389 | 58428.7841796875 | 50593.5160522461 |
| DG(34:1) | 157372.7431030273 | 154359.1176350912 | 138087.2218322757 |
| DG(32:1) | 70186.57329559326 | 79681.40199279784 | 71990.27125295006 |

## Slide 11
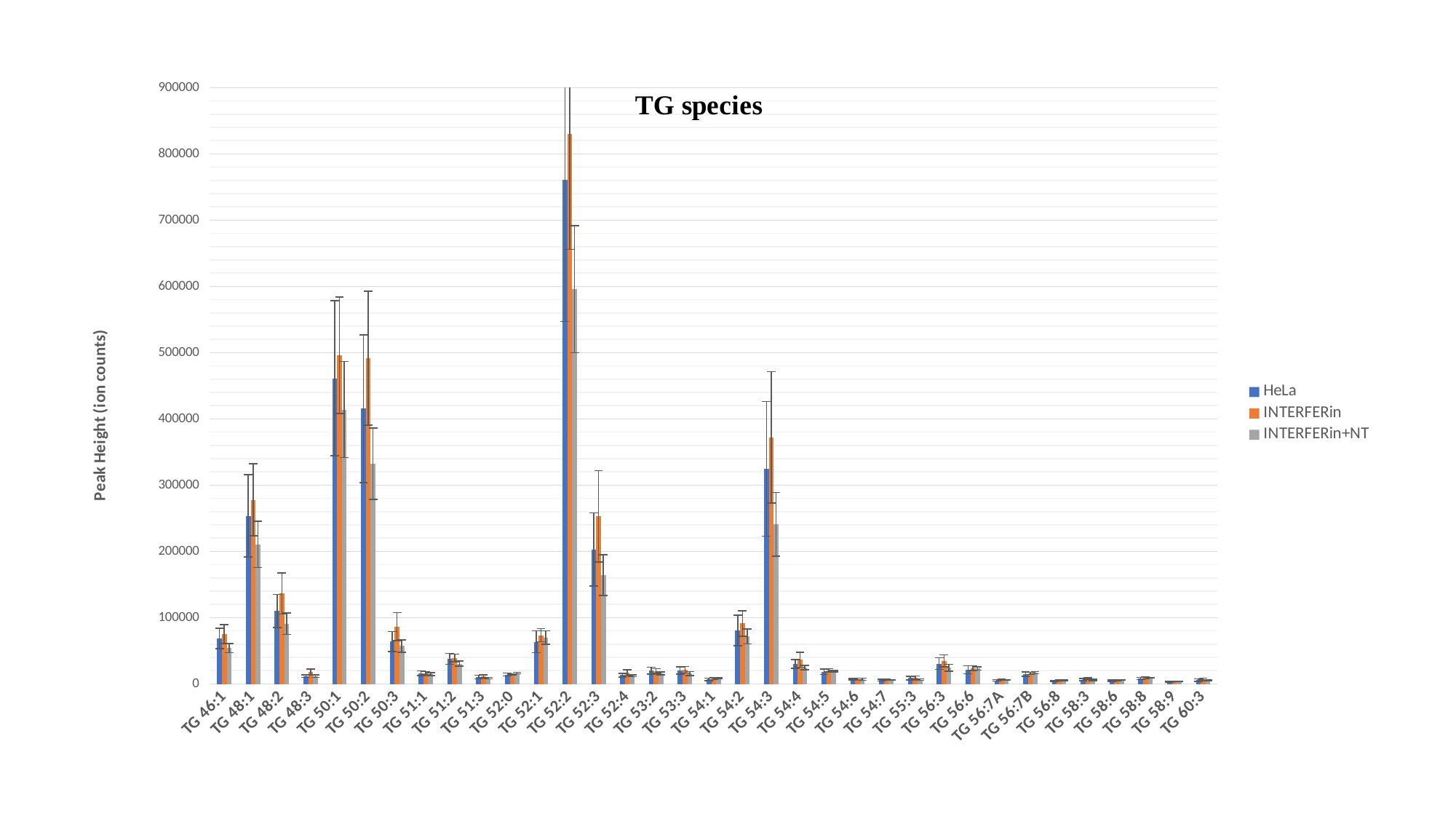

### Chart
| Category | HeLa | INTERFERin | INTERFERin+NT |
|---|---|---|---|
| TG 46:1 | 68781.11573572573 | 75045.8158448428 | 54227.28499889242 |
| TG 48:1 | 253600.2130275305 | 277928.5188726824 | 210682.8126025643 |
| TG 48:2 | 110219.7763704872 | 136390.5211422225 | 90745.58756753795 |
| TG 48:3 | 11856.05468750001 | 17803.45370229086 | 11841.74103546142 |
| TG 50:1 | 461485.0277918527 | 496107.0037145636 | 414203.3017873568 |
| TG 50:2 | 415524.4008035432 | 491516.4257132841 | 332399.1217627073 |
| TG 50:3 | 63896.82708764628 | 86320.51855781223 | 57155.72445892036 |
| TG 51:1 | 16137.66314317915 | 16377.72428621615 | 15003.80213212213 |
| TG 51:2 | 37875.31226288962 | 39093.60943173175 | 30526.25087774449 |
| TG 51:3 | 10420.00084187469 | 11634.75302046701 | 8689.41239930027 |
| TG 52:0 | 14141.53051757815 | 13912.38960774741 | 16479.44352213545 |
| TG 52:1 | 63546.28982128559 | 73506.38902721969 | 69973.83755349135 |
| TG 52:2 | 761021.7557538046 | 829914.4317436917 | 595580.5770082562 |
| TG 52:3 | 202965.7173756722 | 252991.8295535977 | 164156.9965675829 |
| TG 52:4 | 13274.5949910482 | 16847.58723958335 | 12224.03751627605 |
| TG 53:2 | 20000.30735112885 | 19553.87074856168 | 16034.515279296 |
| TG 53:3 | 20281.63812043098 | 21555.64126650196 | 15522.38751631253 |
| TG 54:1 | 6879.886515291225 | 7944.669599119105 | 8737.9475708185 |
| TG 54:2 | 80771.8978829325 | 91203.0393942428 | 71618.81778115685 |
| TG 54:3 | 324718.1391221899 | 372273.2845483924 | 240794.3019074053 |
| TG 54:4 | 30004.3528846593 | 36569.92947961866 | 24854.4039132542 |
| TG 54:5 | 18312.60518391932 | 20450.83760579432 | 19025.71539306642 |
| TG 54:6 | 6928.9013671875 | 7653.17138671875 | 7138.794514973961 |
| TG 54:7 | 5697.563130696614 | 6879.090881347656 | 5973.791168212891 |
| TG 55:3 | 8837.633600870766 | 9554.9148050944 | 6493.149454752605 |
| TG 56:3 | 30710.08902017283 | 34837.64165427602 | 24342.61725675632 |
| TG 56:6 | 21556.26265462242 | 23873.77152506513 | 23390.00606282555 |
| TG 56:7A | 5428.74126180013 | 6166.026021321617 | 5871.298695882163 |
| TG 56:7B | 14735.46796671548 | 16069.7894897461 | 16965.50313822429 |
| TG 56:8 | 4443.959411621095 | 5723.558186848958 | 5294.491353352868 |
| TG 58:3 | 6454.14870198568 | 7828.737927754722 | 5897.001416524251 |
| TG 58:6 | 4903.431172688803 | 5270.772298177087 | 5683.31656901042 |
| TG 58:8 | 8396.203206380218 | 9336.000386556 | 9291.768961588541 |
| TG 58:9 | 3285.018074035643 | 3434.158589680988 | 3918.733540852867 |
| TG 60:3 | 5695.396003827281 | 7189.229938466383 | 5829.670812018661 |
